# Gene Regulatory Network Inference of Immunoresponsive Gene 1 (*IRG1*) Identifies Interferon Regulatory Factor 1 (*IRF1*) as Its Transcriptional Regulator in Mammalian Macrophages

**DOI:** 10.1371/journal.pone.0149050

**Published:** 2016-02-12

**Authors:** Aravind Tallam, Thaneer M. Perumal, Paul M. Antony, Christian Jäger, Joëlle V. Fritz, Laurent Vallar, Rudi Balling, Antonio del Sol, Alessandro Michelucci

**Affiliations:** 1 Luxembourg Centre for Systems Biomedicine, University of Luxembourg, Esch-sur-Alzette, Luxembourg; 2 Genomics Research Laboratory, Luxembourg Institute of Health, Luxembourg, Luxembourg; 3 NORLUX Neuro-Oncology Laboratory, Luxembourg Institute of Health, Luxembourg, Luxembourg; University of São Paulo, BRAZIL

## Abstract

Immunoresponsive gene 1 (*IRG1*) is one of the highest induced genes in macrophages under pro-inflammatory conditions. Its function has been recently described: it codes for immune-responsive gene 1 protein/*cis*-aconitic acid decarboxylase (IRG1/CAD), an enzyme catalysing the production of itaconic acid from cis-aconitic acid, a tricarboxylic acid (TCA) cycle intermediate. Itaconic acid possesses specific antimicrobial properties inhibiting isocitrate lyase, the first enzyme of the glyoxylate shunt, an anaplerotic pathway that bypasses the TCA cycle and enables bacteria to survive on limited carbon conditions. To elucidate the mechanisms underlying itaconic acid production through *IRG1* induction in macrophages, we examined the transcriptional regulation of *IRG1*. To this end, we studied *IRG1* expression in human immune cells under different inflammatory stimuli, such as TNFα and IFNγ, in addition to lipopolysaccharides. Under these conditions, as previously shown in mouse macrophages, IRG1/CAD accumulates in mitochondria. Furthermore, using literature information and transcription factor prediction models, we re-constructed raw gene regulatory networks (GRNs) for *IRG1* in mouse and human macrophages. We further implemented a contextualization algorithm that relies on genome-wide gene expression data to infer putative cell type-specific gene regulatory interactions in mouse and human macrophages, which allowed us to predict potential transcriptional regulators of *IRG1*. Among the computationally identified regulators, siRNA-mediated gene silencing of interferon regulatory factor 1 (*IRF1*) in macrophages significantly decreased the expression of IRG1/CAD at the gene and protein level, which correlated with a reduced production of itaconic acid. Using a synergistic approach of both computational and experimental methods, we here shed more light on the transcriptional machinery of *IRG1* expression and could pave the way to therapeutic approaches targeting itaconic acid levels.

## Introduction

Immune cells specifically respond to various inflammatory environments based on the nature and type of external stimuli. Macrophages trigger defensive pathways upon stimulation of pattern-recognition receptors, such as toll-like receptors (TLRs) [1,2]. Previous studies have shown that mouse macrophages under pro-inflammatory conditions, such as bacterial infections or lipopolysaccharide (LPS) stimulation, highly express immunoresponsive gene 1 (*Irg1*) [3,4]. Recently, we demonstrated the active role of *IRG1* in antimicrobial responses linking metabolism to immunity [5]: immune-responsive gene 1 protein/*cis*-aconitic acid decarboxylase (IRG1/CAD) catalyzes the decarboxylation of cis-aconitic acid to itaconic acid (also known as methylenesuccinic acid) during the TCA cycle. Itaconic acid is an organic compound that inhibits isocitrate lyase, the first enzyme of the glyoxylate shunt, a savior pathway for bacterial growth under nutrient-deprived conditions. For the first time, we also demonstrated *IRG1* expression and itaconic acid production in LPS-treated human peripheral blood mononuclear cells (PBMCs)-derived macrophages [5]. Thus, IRG1/CAD contributes to the host immune response against bacterial invasion providing an additional support to the innate immune system.

In addition to our findings, *Irg1* was also previously shown to be expressed in mouse macrophages under different TLR ligand stimulations [3,6] and microbial infections [4,7]. High up-regulation of *Irg1* was observed in the lungs of mice when infected with influenza A virus, thus showing its induction also under viral infections [8]. In a different context, *Irg1* was found to be highly expressed in the uterine luminal epithelium of the mouse during the early stages of pregnancy due to the synergistic regulation by progesterone and estradiol mediated by the protein kinase C pathway [9,10]. *IRG1* was also reported to be highly expressed in PBMCs of septic patients where it fosters endotoxin tolerance by enhancing A20 expression via reactive oxygen species (ROS) signaling [11]. Apart from mouse and human, *IRG1* is also expressed in other species under microbial infections. In zebrafish (*Danio rerio*), when infected with *Salmonella* species, *irg1* is specifically expressed by macrophage-lineage cells and is cooperatively regulated by glucocorticoid and JAK/STAT signaling pathways [12]. Furthermore, it was shown that irg1 is a key component responsible for the production of mitochondrial ROS augmenting the bactericidal activity of macrophages. Hence, in zebrafish, IRG1/CAD additionally contributes to immune responses by the production of ROS [12]. Taken together, these findings demonstrate a pivotal role of IRG1/CAD in the immune metabolism axis connecting the immune system with cellular metabolism through the production of itaconic acid and ROS.

Despite the profound biological importance of IRG1/CAD, the molecular mechanisms that induce *IRG1* expression have not yet been investigated. The regulation of *Irg1* expression was reported on several findings, which are rather contrasting, such as *de novo* protein synthesis independent [3] and dependent [13], MyD88-independent [14] and dependent [7], TRIF-independent [15], TLR2- and TLR4-independent [13]. Interestingly, *Irg1* was highly induced when stimulated with IFNγ alone or in combination with TNFα and it was shown that the vast majority of the murine IRG1/CAD protein was found in the mitochondrial fraction [6]. All the above experiments differ by cell types, the nature of the stimuli and perturbing compound concentrations. It is highly likely that *IRG1* is regulated by a complex transcriptional machinery responding to cellular environments and external stimuli. Hence, it is important to unravel its transcriptional machinery to understand its role and expression under specific inflammatory conditions.

Gene regulatory networks (GRNs) capture the dependency between transcription factors, genes, proteins and small molecules underlying cellular processes [16,17]. Inferring regulatory interactions linked to *IRG1* can contribute to identify the major regulatory elements involved in the induction of *IRG1*. There are three main GRN inference approaches, which are widely used. The first is based on existing literature information which use natural language processing algorithms and manual curation to mine scientific articles [18–20]. The second approach is purely data-driven without any prior information. These category of methods infer the regulatory networks directly from multi-omics data based on the underlying conditional dependency structures such as co-expression [16], mutual information [21] or regression [22,23] among the genes. The third category of methods uses predictive modeling and experimental approaches, such as ChIP-Seq, to infer transcription factor—DNA (TF-DNA) binding sites (TFBS) and motifs [24,25].

All the three category of methods have their own advantages and disadvantages, while some require high dimensional data (i.e. high number of replicates and/or perturbation conditions), others are skewed with most studied interactions or biological processes. However, due to the course of dimensionality, most of them suffer from a high number of false positive interactions, thus making the subsequent network analysis of GRNs more elusive. To this end, in this context, we used an integrated algorithm, which leverages on the information from all the three different category of methods (i.e. literature, TFBS and data-driven) to infer the regulatory network of *IRG1*. This algorithm, with some minor modifications from [26], is used to infer the GRN of *IRG1* from literature and TFBS, and to prune inconsistent interactions by contextualizing the model to predict differential expression from genome-wide expression arrays. Putative transcriptional regulators of *IRG1* were hypothesized from the resulting GRN and tested using siRNA-mediated gene silencing experiments in mouse and human macrophages under LPS stimulation.

## Materials and Methods

### Cell culture and stimulations

The murine RAW264.7 macrophage cell line [27] was cultured in DMEM medium (Invitrogen, Life Technologies, Carlsbad, California) with 10% Fetal Bovine Serum (FBS), 0.1mg/mL streptomycin (Invitrogen, Life Technologies, Carlsbad, California) and 100U/mL penicillin (Invitrogen, Life Technologies, Carlsbad, California). For the experiments, lipopolysaccharide (LPS from *Escherichia coli* 055:B5, Sigma) was used at a final concentration of 10ng/mL.

Primary monocytes were extracted from the blood samples of anonymous healthy male donors, donated by the Luxembourgish Red Cross (http://www.croix-rouge.lu/). Human blood samples in the present study were obtained under a mutual agreement between the University of Luxembourg and the Luxembourgish Red Cross for blood donation to non-therapeutic purposes. The institutional review board waived the need for consent. The Comité National d’Ethique de Recherche (CNER) (http://www.cner.lu/) approved this study. The components of the blood (peripheral blood mononuclear cells—PBMCs -, plasma and erythrocytes) were separated by Ficoll density gradient separation. For this purpose, the blood was diluted 1:1 with phosphate buffered saline (PBS, Invitrogen, Life Technologies, Carlsbad, California) in falcon tubes and was transferred to Leucosep tubes (Greiner bio one, Kremsmünster, Austria) filled with 15ml of Ficoll (VWR, Radnor, Pennsylvania). After a 10 minute centrifugation (1000 g at room temperature without break), the PBMCs layer was collected and the CD14+ monocytes were isolated by using the MACS® technology (magnetic separation) from Miltenyi Biotec (Bergisch Gladbach, Germany). PBMCs were mixed with 200μL of CD14 MicroBeads and incubated for 30min at 4°C on a rotating platform followed by magnetic separation using LS-column. Isolated CD14+ monocytes were plated at 4x10^6^ cells per well in 6-well plates (or 2x10^6^ cells per well in 12-well plates, 1x10^6^ cells per well in 24-well plates, 2.5x10^5^ cells per well in 96-well plates) and differentiated for 11 days into macrophages using RPMI 1640 medium (VWR, Radnor, Pennsylvania) supplemented with 10% human serum, off the clot, type AB (A&E Scientific, PAA, Pasching, Austria, lot number: C02108-1021), 0.1mg/mL streptomycin, 100U/mL penicillin and 0.1mM L-glutamine (Invitrogen, Life Technologies, Carlsbad, California). During the differentiation, the medium was replaced with fresh medium on day 4 and day 7. Alternatively, CD14+ monocytes were plated at 2x10^6^ cells per well in 12-well plates and differentiated for 8 days into dendritic cells using human serum supplemented with 20ng/ml of both granulocyte-macrophage colony-stimulating factor (GM-CSF, R&D Systems Europe Ltd., United Kingdom) and interleukin-4 (IL-4, R&D Systems Europe Ltd., United Kingdom). For the experiments, LPS was used at 10μg/mL, while tumor necrosis factor alpha (TNFα, R&D Systems Europe Ltd., United Kingdom) and interferon gamma (IFNγ, R&D Systems Europe Ltd., United Kingdom) were used at a final concentration of 50ng/mL.

### RNA extraction, reverse transcription and real-time PCR

Total RNA was purified from cultured cells using the Qiagen RNeasy Mini Kit (Qiagen) according to manufacturer’s instructions. First-strand cDNA was synthesized from 0.5 to 2μg of total RNA using SuperScript III (Invitrogen) with 1μL (50μM)/reaction oligo(dT)20 as primer. Individual 20μL SYBR Green real-time PCR reactions consisted of 2μL of diluted cDNA, 10μL of 2×iQ SYBR Green Supermix (Bio-Rad), and 0.5μL of each 10μM optimized forward and reverse primers in 7μL RNase free water. Primer sequences designed using Beacon Designer software (Bio-Rad), provided by Eurogentec, or directly designed by Thermo Scientific and are shown in S1 Table. For the human Irg1 primers, the NCBI/Primer-BLAST tool available at http://www.ncbi.nlm.nih.gov/tools/primer-blast/ was used. The PCR was carried out on a Light Cycler 480 (Roche Diagnostics), using the following program: 10min at 95°C and 40 cycles of 30s at 95°C, 30s at 60°C and 30s at 72°C followed by 10s 70–95° melting curves. All experiments, including three no template controls, were performed in triplicates for each sample. For normalization, L27 was amplified simultaneously.

### Transfection experiments

The ON-TARGETplus SMARTpool, containing four different siRNA sequences specifically targeting each of murine *Irf1* (siRNA *Irf1*, L-046743-01-0005), murine *Cebpb* (siRNA *Cebpb*, L-043110-00-0005), human *IRF1* (siRNA *IRF1*, L-011704-00-0005) and the corresponding non-targeting control (siRNA NEG, D-001810-10-05), were designed and synthesized by Thermo Scientific Dharmacon.

Murine RAW264.7 macrophages were transfected with Amaxa 4D Nucleofector device, X-unit (Lonza) using the Amaxa SG cell line 4D Nucleofector Kit for THP-1 cells according to the manufacturer’s instructions. Briefly, transfection with siRNA complexes was carried out from pelleted and resuspended cells (1x10^6^ cells per condition). Transfection reagent and siRNA were prepared according to the manufacturer’s instructions (Amaxa). Specific siRNAs were added at a final concentration of 100nM. After nucleofection using the program “RAW264.7 (ATCC)”, the cells were seeded at a density of 1x10^6^ cells per well in 12-well plates in DMEM supplemented with 10% FBS and incubated for 24h.

Human PBMCs-derived macrophages were transfected using the Amaxa 4D Nucleofector device, Y-unit, which was specifically designed for transfection of adherent cells. For these experiments, cells were seeded at 1x10^6^ cells in 24-well plates for 11 days and the medium was then replaced with nucleofection reagent and specific siRNAs (2μM) according to the manufacturer’s protocol. The reagent solution was removed and the medium was added to the cells which were then incubated for 24h and stimulated with LPS.

### Immunofluorescence and automated image analysis

For immunofluorescence the cells were grown on CellCarrier 96 well plates (Perkin Elmer), washed with PBS, and fixed with 4% paraformaldehyde (pH 7.4) for 30min at ambient temperature. After washing in PBS, the cells were permeabilized for 5min with 0.5% Triton X-100 in PBS. Permeabilization was followed by 3 x 10min washing steps in PBS. For blocking, samples were incubated with 5% goat serum in PBS for 1h at room temperature. Primary antibodies against IRG1/CAD (Anti-IRG1 antibody produced in rabbit, Sigma, 1/200) and a mitochondrial surface antigen (MAB1273, Millipore, 1/100) were diluted in PBS + 1% BSA and bound for 1h at room temperature. After three washing steps in PBS, secondary antibodies (A-21428, A-11001, Invitrogen, both 1/500) were added and incubated at dark for 1h at room temperature. For staining of nuclei and stabilization of fluorescent signals the samples were covered with Fluoroshield mounting medium containing DAPI (F6057, Sigma). Image acquisition started after 10min of incubation.

Image stacks with 11 planes were acquired on a confocal Opera QEHS High Content screening microscope (Perkin Elmer), using a 60x water immersion objective (NA 1.2). The antibody-labeled mitochondrial channel was excited with a 488nm laser and detected with a 520/35 band-pass filter. Antibody-labeled IRG1/CAD channel was excited with a 561nm laser and detected with a 600/40 band-pass filter. DAPI was excited with a 405nm laser and detected with a 450/50 band-pass filter.

Automated image analysis was performed in Matlab 2015a. Nucleus segmentation was following the rule that pixels of low pass filtered DAPI images, convolved with a gaussian filter of size 20 and sigma 5 have to be at least 25% brighter within nuclei as compared to local surroundings as defined by an average filter of size 100. The minimum size of nuclei was set to 1000 pixels. For the detection of cell covered regions, the three channels were summed up and low pass filtered with a gaussian filter of size 50 and sigma 20. Resulting images were thresholded by the first quartile of pixel values. To detect single cell areas, a watershed algorithm was applied to the euclidian distance transform of the nucleus mask. Mitochondria were segmented via a combination of local thresholding and segmentation of non-uniformly corrected mitochondria images. According to the local thresholding algorithm, mitochondrial pixel assignment requires a raw mitochondrial image, low pass filtered with a gaussian filter of size 5 and sigma 2 to be brighter than the same image convolved with an average filter of size 5. For non-uniform correction mitochondria channel images average filtered with a structuring element of size 50 were subtracted from original mitochondria images. For segmentation these corrected images were thresholded to a pixel intensity of 10. For connected components confirmed by both rules the minimum volume was set to 10 pixels. IRG1/CAD positive pixels were defined by the same algorithm as mitochondrial pixels, in this case applied to the IRG1/CAD channel. Subcellular localization of IRG1/CAD was evaluated by computing single cell proportions of IRG1/CAD positive pixels in nuclei and mitochondria.

### Protein extraction

Cells were washed with ice-cold PBS after removing the medium. Proteins were extracted using Mammalian Protein Extraction Reagent (M-PER, Thermo Scientific) which includes lysis buffer and a protease inhibitor. A volume of 150μL/well of M-PER complete reagent was added to lyse the cells. Cells were then scraped out from the plates and the lysate was shaken at 4°C for 20min at maximum speed. The lysate was then centrifuged at 4°C for 5min at maximum speed. The supernatant was collected and stored at -80°C. The extracted protein samples were then quantified using a Bradford assay and the measurements were used for subsequent western blotting.

### Western blotting

Heat-denatured protein samples (20μg) were separated on a 10% SDS-polyacrylamide gel electrophoresis followed by transfer to nitrocellulose membranes 0.2μm (Sigma). Affinity-purified goat anti-mouse IRF1 antibody (catalogue #: AF4715) and rabbit anti-goat IgG secondary antibody (catalogue #: HAF017) were obtained from R&D Systems Europe Ltd., United Kingdom, while rabbit anti-human IRG1 antibody (catalogue #: HPA040143) and normal rabbit IgG (catalogue #: sc-2027) were purchased from Sigma and Santa Cruz Biotechnology, respectively. Goat anti-β-actin (catalogue #: sc-1616) and anti-goat secondary antibodies (catalogue #: RPN1025) were purchased from Santa Cruz Biotechnology and GE Healthcare, respectively. After blocking with 5% (wt/vol) dry milk in PBS, the membrane was incubated overnight at 4°C with primary antibody in 1% BSA/PBS (dilution 1:2500 for IRF1 and IRG1) on a rotating platform. After three washing steps with PBS containing 0.1% Tween-20, the membrane was incubated with secondary antibodies (dilution 1:5000) coupled to horseradish peroxidase and revealed by chemiluminescence using the Amersham ECL detection reagents (GE Healthcare) and ODISSEY imaging system.

### Metabolite extraction

Cells seeded in 6-well plates were washed with 1mL of saline solution (0.9% NaCl) and quenched with 0.4mL cold methanol. After adding an equal volume of cold water, cells were collected with a cell scraper and transferred in tubes containing 0.4mL cold chloroform. The extracts were vortexed at 1400rpm for 20min at 4°C and centrifuged at 13500rpm for 5min at 4°C. A volume of 0.3mL of the upper aqueous phase was collected in specific GC glass vials and evaporated under vacuum at -4°C using a refrigerated CentriVap Concentrator. Extractions of metabolites from cells grown in 12-well plates were performed using half of the volumes. The interphase was centrifuged with 50μL cold methanol at 13500rpm for 5min at 4°C. When needed, the pellet was stored at -80°C for subsequent RNA isolation.

### Gas chromatography/mass spectrometry (GC/MS) analysis

Metabolite derivatization was performed using a multi-purpose sampler (Gerstel). Dried samples were dissolved in 15μL pyridine, containing 20mg/mL methoxyamine hydrochloride, at 40°C for 60 minutes by shaking. After adding 15μL N-methyl-N-trimethylsilyl-triflouroacetamide (MSTFA), samples were incubated at 40°C for 30min with continuous shaking. GC-MS analysis was performed by using an Agilent 7890A GC coupled to an Agilent 5975C inert XL MSD. A sample volume of 1μL was injected into a split/splitless inlet operating in splitless mode at 270°C. The gas chromatograph was equipped with a 30m Agilent J&W DB-35MS capillary column + 5m DuraGuard capillary in front of the analytical column. Helium was used as carrier gas with a constant flow rate of 1.2ml/minute. The GC oven temperature was held at 90°C for 1min and then increased to 320°C at 15°C/minute. The final temperature was held for 8min. The transfer line temperature was set constantly to 280°C. The MSD was operating under electron ionization at 70eV. The MS source was held at 230°C and the quadrupole at 150°C. The GC-MS was operated in Selected Ion Monitoring (SIM) mode (m/z 215.1, m/z 230.1, m/z 259.1). The total run time of one sample was 24.3min.

All GC-MS chromatograms were processed using MetaboliteDetector [28] for targeted data analysis. The software package supports automatic deconvolution of all mass spectra. The obtained mass spectra were matched against a reference library (including the mass spectrum of the authentic standard “itaconic acid”). Compounds were annotated by retention time and mass spectrum (overall similarity: 0.95 or higher). For quantification, fragment ion m/z 259 was used.

### RNA isolation, microarray hybridisation and data analysis

Total RNA from PBMCs-derived macrophages was extracted using TRIzol reagent (Invitrogen) while total RNA from RAW264.7 cells was harvested using the Qiagen RNeasy Mini Kit (Qiagen), according to the manufacturer’s protocols. RNA purity and integrity were monitored using NanoDrop® ND-1000 spectrophotometer and Agilent 2100 Bioanalyzer with RNA 6000 Nano assay kit. Only RNAs with no sign of contamination or marked degradation (RIN > 9) were considered good quality and used for further analysis. GeneChip Human Gene 1.0ST and GeneChip Mouse Gene 2.0ST Arrays (Affymetrix) were used to determine the genome-wide expression profiles of PBMCs-derived macrophages and RAW264.7 cells, respectively. Total RNAs (250ng) were processed using the Affymetrix Whole Transcriptome (WT) Expression kit according to the manufacturer’s instructions (User manual P/N 4425209 Rev. C 09/2009). Microarrays were hybridized, washed and stained using the Affymetrix GeneChip WT Terminal Labeling and Hybridization kit following the microarrays were washed, stained and scanned according to manufacturer’s standard procedures (User manual P/N 702808 Rev. 6). Genechips were scanned using an Affymetrix GeneChip Scanner 3000 generating CEL files containing hybridization raw signal intensities which were preprocessed and normalized using the GeneChip Robust Multiarray Averaging (GCRMA) algorithm [29] from Bioconductor in R. Redundant probe sets were merged by considering mean values, resulting in a list of unique annotated genes mapped based on Entrez gene identifiers. Using the limma package and the eBayes function from Affy library in Bioconductor, genes whose expression values between any two conditions having a difference with a log fold change (logFC) ≥ +/-1 and a p-value<0.01 were considered as differentially expressed genes (DEGs). Microarray expression data are available at Gene Expression Omnibus (GEO) database (http://www.ncbi.nlm.nih.gov/geo/) under the accession number GSE76563.

### Gene regulatory network (GRN) inference

GRN inference was performed using three distinct approaches:

(a) From transcription factor—DNA (TF-DNA) binding models: Upstream regulation of *IRG1* was inferred using MATCH™ algorithm of TRANSFAC^®^ database from the BioBase International Corporation [25,30]. MATCH™ algorithm is a weight-matrix based algorithm which searches for potential transcription factor binding sites (TFBS) on any given genomic sequence using the position weight matrices (PWMs) library from TRANSFAC^®^ database. The PWMs for transcription factors (TF) in TRANSFAC^®^ are constructed based on the consensus of its DNA binding sequences across the genome. Each PWM consists of the nucleotides and their frequency at respective position on the binding sequence. For our analysis, immune cell specific profiles of MATCH^™^ consisting of PWMs of TF that are involved in the immune responses in T-cells, B-cells, mast cells, myeloid cells, natural killer cells and macrophages were used.

Transcription start site (TSS) information of genes from RefSeq and genome sequences of range 2000bp upstream and 1000bp downstream with respect to TSS from hg19 (human), mm10 (mouse) from UCSC website were obtained. Taking these sequences as input, MATCH^™^ algorithm searches for potential binding sites of TFs on the sequence using its collection of PWMs. The output consists of information about the transcription factor, binding position, genomic strand and the two quality scores (Core and Matrix similarity), sequence motif of each binding site found. Only the transcription factor binding predictions that have both the similarity scores ≥ 0.90 were considered for further analysis (S2 Table).

(b) From published literature: On the other hand, downstream regulation of LPS stimulus in macrophages was inferred using Pathway Studio Desktop 10 from Elsevier [20]. Pathway Studio [20] is the knowledge base of ontologies, taxonomies and biological relationships derived based on the text mining algorithms and the expert curation of published scientific literature. MedScan® text mining technology was used to scan all the PubMed abstracts (≈30 million) and relevant sentences were collected based on the manually curated dictionaries with the synonyms of biological terms. For this network, the extraction of published literature was restricted as follows: species: human/mouse; cell type: macrophages; interactions: direct regulations in the network building settings of the Pathway Studio software.

(c) Combining and contextualizing literature and TFBS networks: By virtue, the GRN inferred above are from different cell, tissue and/or organism. Also, for some of the TF-DNA predictions and literature inferences, the mode of action is unspecified (i.e. activation or inhibition). Therefore, in order to find context specific interactions (i.e. that are specific to *IRG1* expression in mouse macrophages and/or human PBMCs), we prune the inconsistencies in the inferred GRN with an improved version of the previously developed contextualization algorithm [31]. In gist, using feedback regulations as basic building blocks, this algorithm tries to predict differential expression from genome-wide arrays, using a boolean modeling framework, by removing as well as assigning mode of actions to the interactions in the GRN [32]. This method was implemented in Matlab using the genetic algorithm (ga) function with the assumption that each cell phenotype represents a stable steady state (attractor) of the network. The pbn-matlab-toolbox (http://code.google.com/p/pbn-matlab-toolbox/downloads/list) with the synchronous updating scheme was employed in this algorithm for the Boolean simulation.

### Prioritising transcriptional regulators

In order to identify the transcriptional regulators for *IRG1* under LPS stimulation, the contextualised GRN was used to hypothesize experimentally testable predictions. All simple paths connecting LPS stimulation to *IRG1* from the inferred GRN were computed using the graph k shortest paths algorithm of Matlab. These paths provide the basis set of putative regulators and pathways for translating the LPS signal to induce *IRG1*. Further, using the simple paths, the importance of TFs were established using a gene essentiality metric score. This score defines the importance of a particular gene in propagating the signal from the ligand stimulus to the target gene. The score ranges from 0 to 1 with 1 being the most essential gene and 0 being non-essential. However, the score cannot be 0 for any gene as all the genes are at least participating in one of the paths between LPS-*IRG1*. These genes were then ranked according to their essentiality metric in these paths. The score was calculated according to the formula:

$$EM_{j}=\frac{N_{TP}-N_{jP}}{N_{TP}}$$

**Fig 1:**
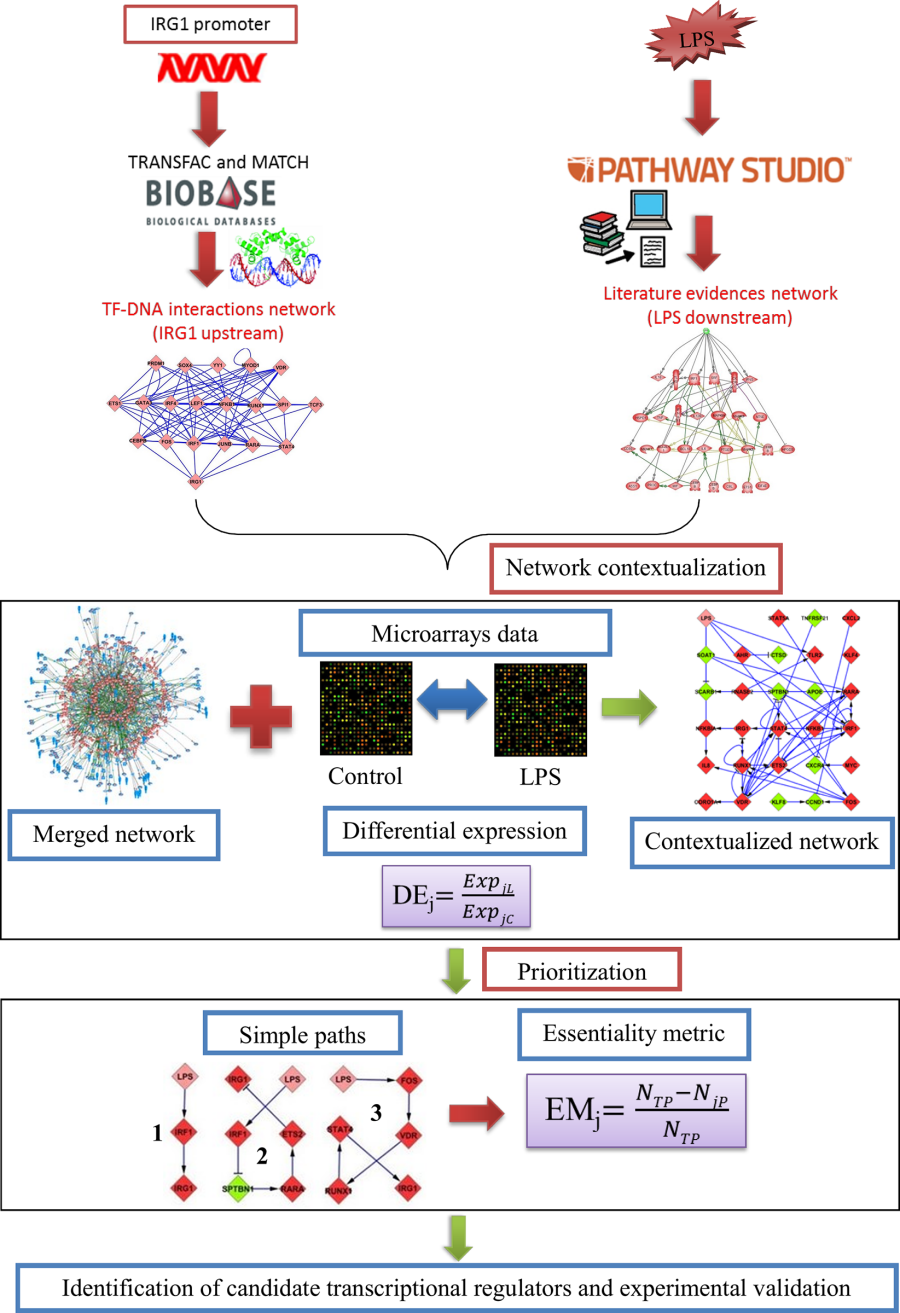
Workflow for the identification of transcriptional regulators for a specific gene. The scheme shows the workflow of the different steps followed to identify potential transcriptional regulators for a given gene under LPS exposure. The upstream network was initially constructed using the PWMs from TRANSFAC^®^ and the prediction algorithm MATCH™. The literature network downstream of LPS was inferred using the Pathway Studio knowledge database. These networks were merged and the merged network was contextualised using the booleanised genome-wide expression data. Finally, the ranking scheme with simple paths and essentiality metric resulted in a set of potential transcriptional regulators which were tested using siRNA mediated gene silencing experiments.

Where EM_j_ denotes the essentiality metric for gene *j*, N_TP_ denotes the number of total paths, N_jP_ denotes the number of paths in which gene *j* is absent. Hence, if the number of paths in which a particular gene is absent is low, its essentiality metric will be high. The top ranked TFs were then chosen to perform siRNA-mediated gene silencing experiments to analyze the effect on *IRG1* expression. Overall, the workflow from the inference of GRNs to validation studies is depicted in Fig 1.

### Statistical analysis

Otherwise mentioned, p-values were calculated according to the Student t-test with the two-tailed distribution assuming two-sample equal variance.

## Results

### *IRG1* is expressed by different immune cells under various inflammatory conditions

**Fig 2:**
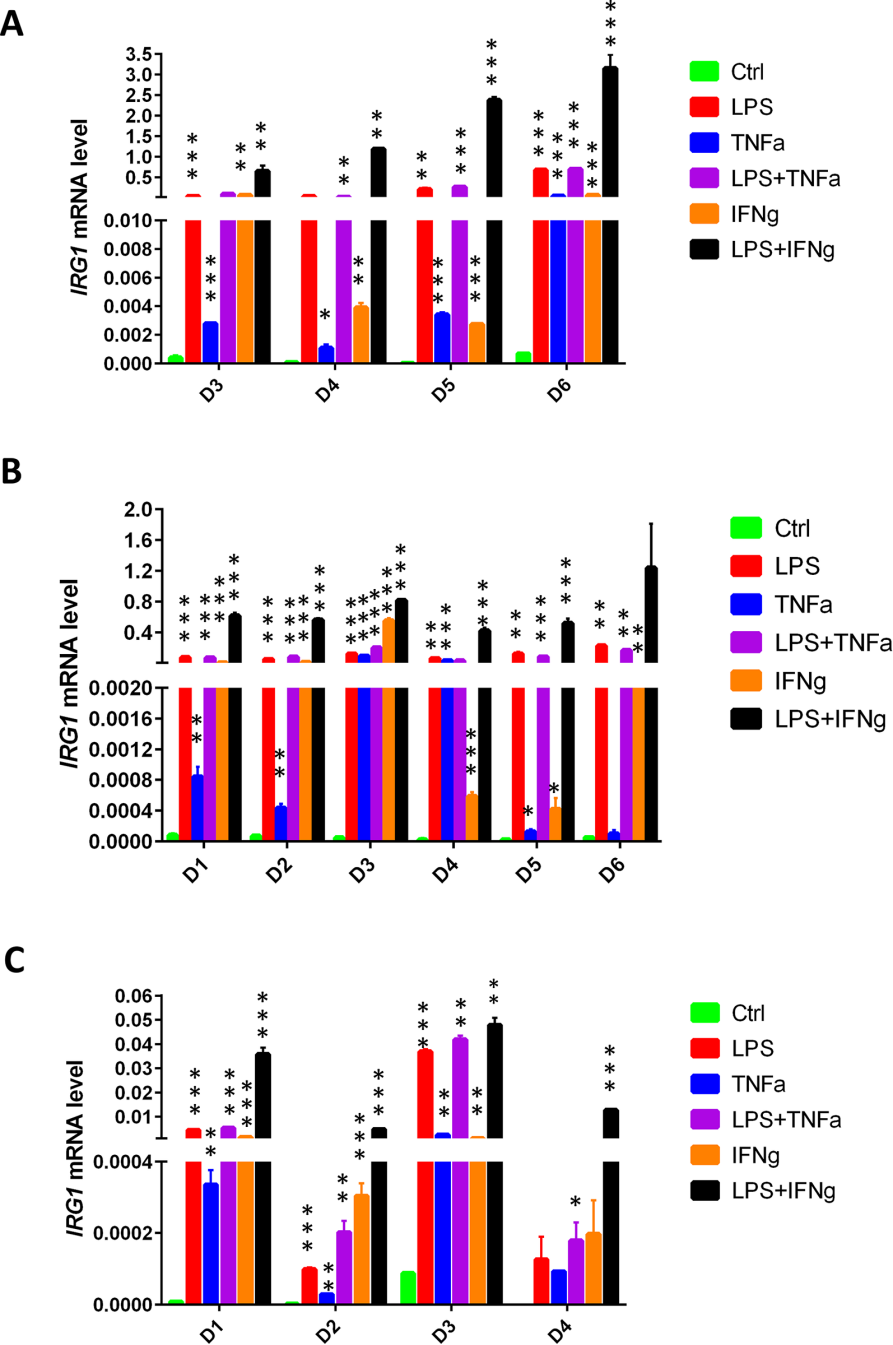
*IRG1* expression in human immune cells under pro-inflammatory conditions. RNA was extracted from (**A**) PBMCs-derived monocytes, (**B**) PBMCs-derived macrophages and (**C**) PBMCs-derived dendritic cells 6 hours after treatment with LPS (10μg/ml), TNFα (50ng/ml), IFNγ (10ng/ml) and LPS together with TNFα or IFNγ in independent donors (D1-D6). The bars show the mean of 3 technical replicates (± SEM) of *IRG1* mRNA levels measured by real-time PCR normalised with L27 as the housekeeping gene. ***p < 0.001, **p < 0.01, *p < 0.05.

We first analysed *IRG1* expression in human PBMCs-derived monocytes (Fig 2A), PBMCs-derived macrophages (Fig 2B) and PBMCs-derived dendritic cells (Fig 2C) under various inflammatory conditions induced either by LPS (10μg/mL), TNFα (50ng/mL), IFNγ (10ng/mL), LPS with TNFα or IFNγ exposures. After 6 hours, the expression levels of *IRG1* were highly increased following the different pro-inflammatory treatments in all the analysed immune cells compared to the untreated cells, with the highest levels obtained after their exposure to LPS in combination with IFNγ (Fig 2). In order to investigate *IRG1* expression over time following a pro-inflammatory stimulus, we analysed its expression levels at different time points following LPS activation. The results show that *IRG1*, similarly to the corresponding mouse gene [5], is expressed at the highest levels at 6–9 hours in both monocytes and macrophages (S1 Fig) and that its expression is already significantly induced after 40 minutes in human PBMCs-derived macrophages as well as after 20 minutes in the murine RAW264.7 macrophage cell line (S2 Fig). Taken together, these results show that *IRG1* is expressed by human monocytes, macrophages and dendritic cells and that it is rapidly induced in both human and murine macrophages under inflammatory conditions.

### IRG1/CAD associates with mitochondria in human macrophages under inflammatory conditions

**Fig 3:**
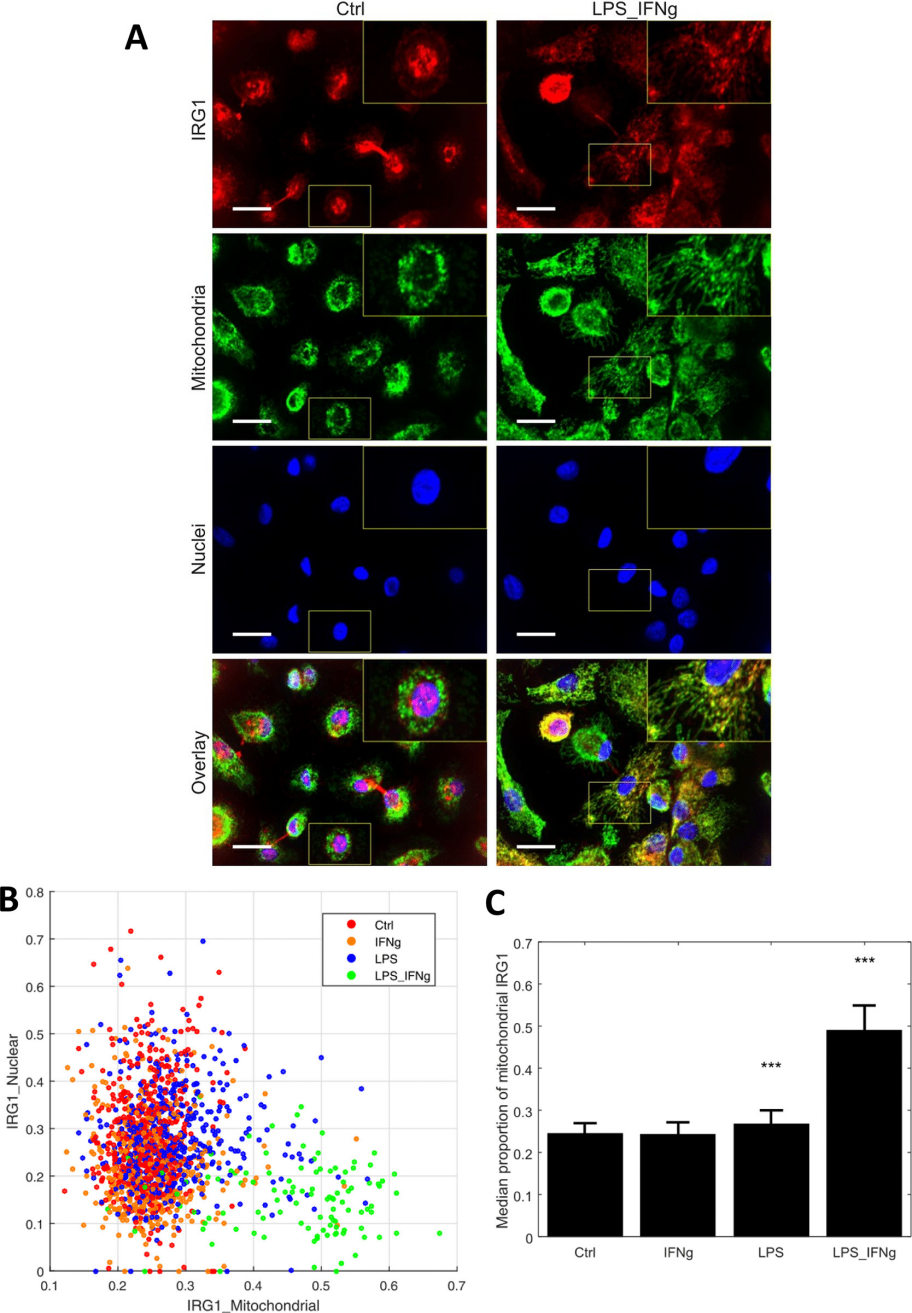
Mitochondrial accumulation of IRG1/CAD under pro-inflammatory conditions. (**A**) Immunofluorescence images show maximum projections from 11 confocal planes. Scale bars indicate 20μm. Additionally magnified image regions and 2x magnified inlets are highlighted with yellow boxes. (**B**) IRG1/CAD was detected in nuclei, mitochondria and cytoplasmic vesicular structures. To quantify mitochondrial accumulation of IRG1/CAD, nuclear and mitochondrial proportions of single cell IRG1/CAD positive pixels were analysed. (**C**) Bars represent the median proportion of mitochondrial IRG1/CAD in untreated cells, LPS, IFNγ and LPS with IFNγ treated cells. Error bars show median absolute deviations. Statistical testing was done via two sided Wilcoxon rank sum tests. ***p < 0.001 (Wilcoxon rank sum test).

It was previously shown that the murine IRG1/CAD protein associates with mitochondria [6]. However, the sub-cellular localization of the corresponding human protein was not described until now. Thus, to elucidate IRG1/CAD compartmentalization in human macrophages, we applied immunofluorescence in combination with confocal microscopy to human PBMCs-derived macrophages under inflammatory conditions. Results showed that, similarly to the murine protein, human IRG1/CAD is accumulating in mitochondria (Fig 3A and 3B). Indeed, treatments with LPS alone or in combination with IFNγ caused significant mitochondrial accumulation of IRG1/CAD (Wilcoxon rank sum test, p < 0.001) (Fig 3C).

### *IRG1* gene regulatory network inference and network contextualisation in mammalian macrophages

Following our results at the gene and protein level, we investigated in more details the transcriptional machinery, which is responsible for *IRG1* expression in both murine and human macrophages. Using BIOBASE resources, such as TRANSFAC^®^ and MATCH^TM^, we inferred the TF-DNA interactions (directed and unsigned) with *IRG1* as the starting node. We adopted a similar approach for both the murine and human genes. The mouse analysis resulted in an *Irg1* upstream network containing 70 nodes and 3936 edges. In parallel, the interactions (signed and directed) related to the biological process of LPS stimulation in macrophages were inferred from Pathway Studio and resulted in a LPS downstream network with 615 nodes and 1278 edges. As expected, we did not obtain *Irg1* as one of the nodes in this network reflecting the lack of specific information about the transcriptional regulation of *Irg1* following an LPS treatment. Taking the union of all the interactions, these two networks were then merged into a single network. The merged network with 663 nodes and 5214 edges established the connection between LPS and *Irg1* with common nodes from both networks.

Similarly to the analysis performed in mouse macrophages, the MATCH^TM^ algorithm with the human *IRG1* promoter sequence and the immune cell specific profile yielded an *IRG1* upstream network with 75 nodes and 4235 edges, which passed the quality threshold. The literature network downstream of LPS inferred from Pathway Studio resulted in a network of 1458 nodes and 2857 edges. As for the mouse analysis, these two networks were then merged with a total of 1490 nodes and 7092 edges.

**Fig 4:**
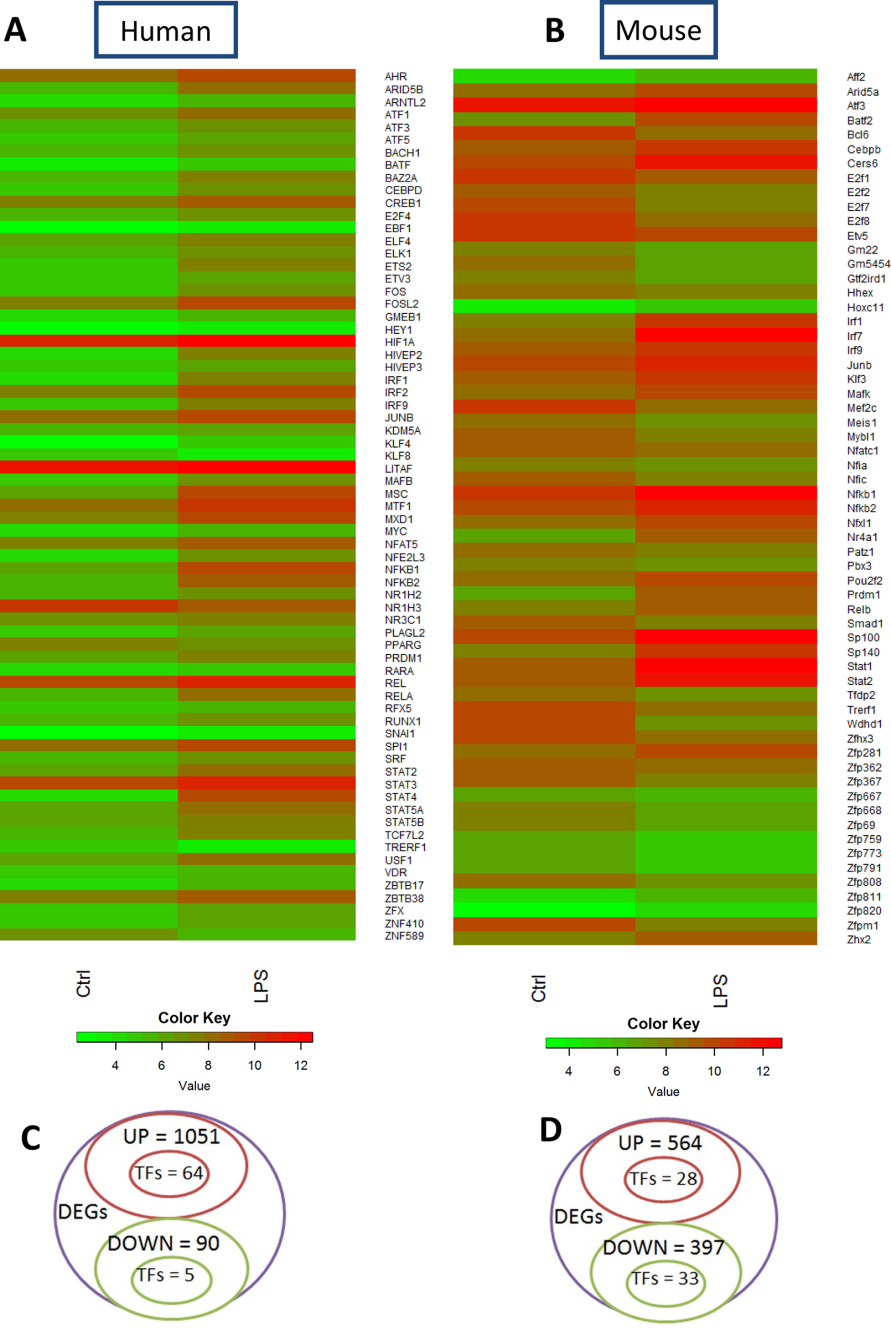
Heatmap of transcription factors and Venn diagrams of microarrays data. (**A, B**) Heatmaps of all the differentially expressed transcription factors (source: Animal TFDB) from (**A**) human and (**B**) mouse microarrays data. (**C, D**) Venn diagrams showing the number of differentially expressed genes and transcription factors under LPS stimulation in (**C**) human PBMCs-derived macrophages and (**D**) mouse RAW264.7 macrophages.

Since the murine and human merged networks were obtained from heterogeneous data, we generated genome-wide gene expression data in the murine RAW264.7 macrophage cell line and human PBMCs-derived macrophages after 6 hours of LPS stimulation for network contextualization. The differentially expressed genes (DEGs) with their respective fold changes and p-values are included in the supplementary material (S3 and S4 Tables). From these genes, we identified the top 100 DEGs (log2 FC≥|1| and p-value <0.01) between untreated and LPS-activated conditions for both mouse RAW264.7 macrophages (S3 Fig) and human PBMCs-derived macrophages (S4 Fig), respectively. The results show that both mouse and human cells are highly affected by LPS displaying a classical pro-inflammatory signature were the classical pro-inflammatory markers, such as IL1β, CCL5 and IL6, are among the top 100 DEGs in both the species. Gene Ontology (GO) analysis reveals up-regulation of biological processes that are reflecting a pro-inflammatory response, such as “Response to molecule of bacterial origin” or “Regulation of cytokine production” in both mouse and human cells (S3 Fig and S4 Fig). Down-regulated biological processes are related to cell cycle in mouse cells (S3 Fig), while in human macrophages they are associated to different metabolic processes, such as lipid metabolism (S4 Fig). In order to focus our analysis on transcriptional regulators, we generated heatmaps showing all the differentially expressed transcription factors under the previous conditions (Fig 4A and 4B). The classical pro-inflammatory-associated transcription factors, such as NFκB, JUNB and IRFs, are highly up-regulated in LPS conditions when compared to untreated cells. In human cells, 1051 genes were up-regulated in LPS conditions when compared to control and, among them, 64 were transcription factors, while a total of 90 genes were down-regulated and 5 were transcription factors (Fig 4C). In mouse macrophages, a total of 564 genes were up-regulated and, among them, 28 were transcription factors, while 397 genes were down-regulated and 33 were transcription factors (Fig 4D).

The relevance of these genome-wide gene expression data to prune the networks allowed us to remove the interactions that were inconsistent with the gene expression data as well as to infer the signs to the previously unsigned interactions from *IRG1* upstream networks. The interactions of LPS with the nodes of this contextualised networks were retrieved from the LPS downstream networks and added to the contextualised networks. The resultant signed and directed mouse network had 40 nodes and 70 edges (S5 Fig), while the human network had 33 nodes and 72 edges (S6 Fig).

### Identification of *IRG1* transcriptional regulators from the contextualised networks

**Fig 5:**
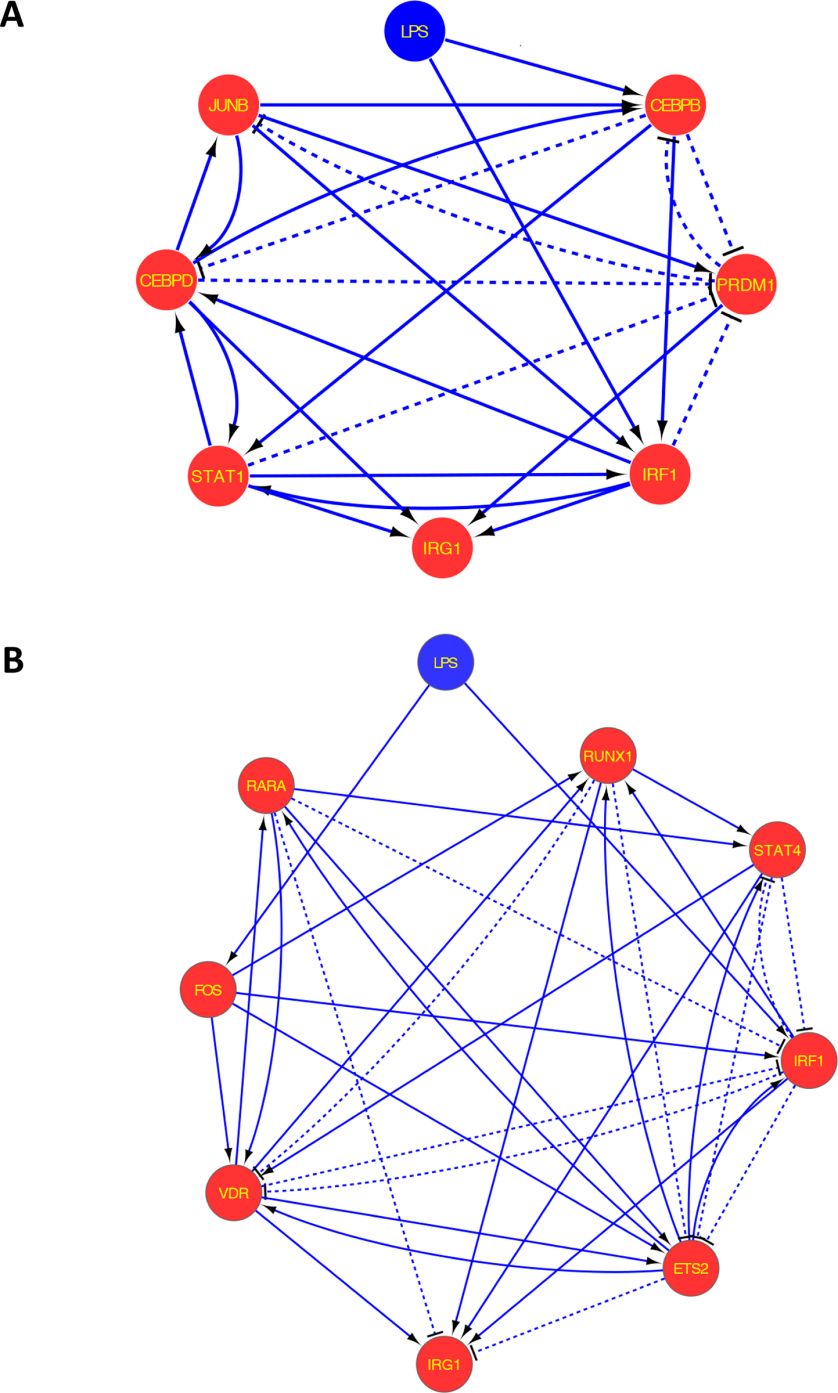
All possible paths from LPS to *IRG1* from the contextualised network. (**A**) Mouse and (**B**) human networks hierarchical layouts after calculating all possible paths between LPS and *IRG1* from the contextualised networks. *IRF1*, interferon regulatory factor 1; CEBPB, CCAAT/enhancer binding protein (C/EBP) beta; CEBPD, CCAAT/enhancer binding protein (C/EBP) delta; STAT1, signal transducer and activator of transcription 1; JUNB, Jun B proto-oncogene; PRDM1, PR domain containing 1 with ZNF domain; STAT4, signal transducer and activator of transcription 4; FOS, FBJ murine osteosarcoma viral oncogene homolog; ETS2, v-ets avian erythroblastosis virus E26 oncogene homolog 2; VDR, vitamin D (1,25-dihydroxyvitamin D3) receptor; RARA, retinoic acid receptor alpha; RUNX1, runt-related transcription factor 1. Solid line: activation; dashed line: inhibition.

From the contextualised networks, we then aimed to identify the potential transcriptional regulators of *IRG1*. For this, all the possible paths that connect LPS and *IRG1* were calculated using the K Shortest Simple (loopless) Paths—“graphkshortestpaths”—implementation from Matlab and the representation of the resultant networks is shown in Fig 5. In the mouse network, *Irg1* has direct connections with the transcriptional regulators IRF1, PRDM1, CEBPD and STAT1, while it has indirect connections with JUNB and CEBPB (Fig 5A). Similarly, in the human network, *IRG1* is directly connected to the transcriptional regulators IRF1, VDR, STAT4, RUNX1, RARA and ETS2, with RARA and ETS2 be predicted to have an inhibitory effect on *IRG1* expression. Moreover, *IRG1* has one indirect interaction with FOS, which, according to the network, transcriptionally regulates *IRG1* in multiple ways (Fig 5B).

Given the topology of these simple paths networks, we then calculated the gene essentiality metric for all the transcriptional regulators to rank their importance in the network (Table 1). The essentiality metric scores were calculated as described in *Materials and Methods*. The total paths represent the number of paths in the simple paths networks from LPS to *IRG1*. The paths present after perturbation of transcriptional regulators is the number of paths where a specific regulator is not present and does not disturb the signal transduction between LPS and *IRG1*. A higher essentiality metric score value corresponds to a higher importance of the specific node between LPS and *IRG1*.

**Table 1:** Transcription factors gene essentiality metric scores.

Transcriptional regulator	Total paths	Paths present after perturbation of a transcriptional regulator	Essentiality metric
**Mouse**			
IRF1	92	14	0.85
CEBPB	92	15	0.84
CEBPD	92	18	0.80
PRDM1	92	22	0.76
STAT1	92	26	0.72
JUNB	92	45	0.51
**Human**			
IRF1	100	27	0.73
ETS2	100	44	0.56
FOS	100	46	0.54
VDR	100	57	0.43
RUNX1	100	65	0.35
RARA	100	70	0.30
STAT4	100	73	0.27

The gene essentiality metric scores were calculated from the mouse and human simple paths networks. Columns description: (1) transcription factor, (2) total number of paths from the simple paths network (***Np***), (3) paths present when a particular transcription factor is perturbed (absent) (***N*j**), (4) essentiality metric score calculated as **EMj** = (***Np*** - ***N*j**)/ ***N*j**.

Based on these scores, we were then interested to investigate the effect of the transcriptional regulator IRF1 on *IRG*1 expression, since from our ranking, IRF1 resulted to be the top scoring transcriptional regulator in both the mouse and human networks.

### Silencing of *IRF1* reduces *IRG1* expression levels in mammalian macrophages

Gene interference mediated by siRNAs is widely used to study the effects caused by gene silencing in functional genomics and therapeutic applications [33,34], hence we adapted this technique to our cellular models. Mouse RAW264.7 cells or human PBMCs-derived macrophages were transfected with siRNA targeting *IRF1* or with a non-targeting siRNA as control. After 24 hours of transfection, mouse cells were stimulated with LPS and RNA was extracted after 2 hours. As a proof of concept, we first silenced *Cebpb*, our second top scoring transcriptional regulator. Indeed, CEBPB was already previously described to be a transcription factor responsible for *irg1* induction in zebrafish [12]. Silencing of *Cebpb* resulted in 55% and 45% decrease of the *Cebpb* mRNA levels when compared to the non-targeting siRNA in unstimulated and LPS-treated cells, respectively (S7 Fig). Correspondingly, *Irg1* expression was decreased by 69% and 16% in *Cebpb* silenced cells as compared to non-targeting siRNA in unstimulated and LPS treated cells, respectively (S7 Fig). Thus, these results confirm the previous findings in zebrafish, showing that CEBPB is a transcriptional regulator of *Irg1* in mouse macrophages, as also predicted by our analysis.

**Fig 6:**
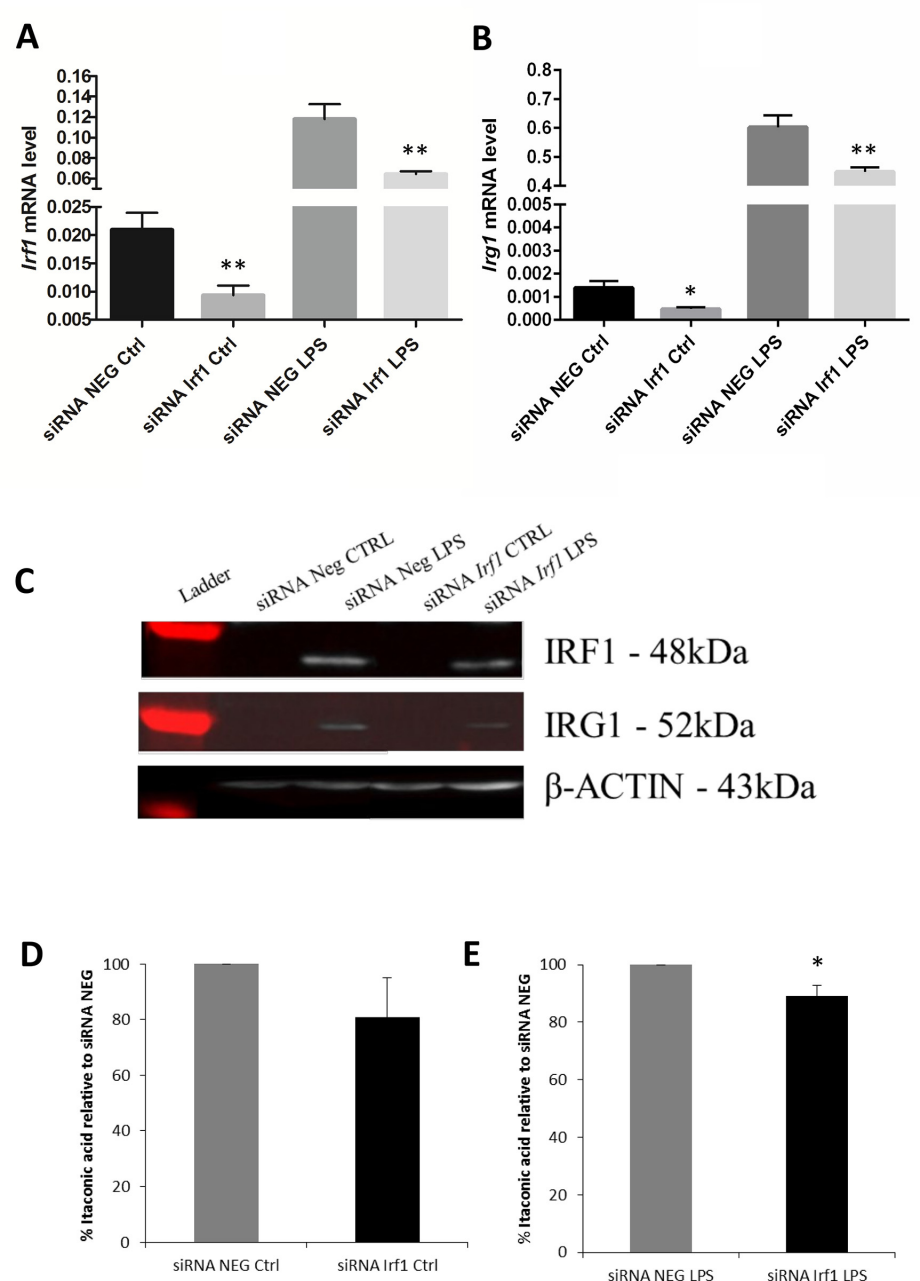
siRNA *Irf1* mediated gene silencing in mouse RAW264.7 macrophages. (**A, B**) RAW264.7 cells were transfected with siRNA negative (siRNA NEG) or siRNA specific to *Irf1* (siRNA Irf1) 24 hours before treatment and RNA was extracted 2 hours after activation with LPS (10ng/ml). The bars show the mean of 3 biological replicates (± SEM) of (**A**) *Irf1* and (**B**) *Irg1* mRNA levels measured by real-time PCR normalised with L27 as the housekeeping gene. **p < 0.01, *p < 0.05. (**C**) Proteins were extracted from transfected cells after 4 hours of LPS stimulation. Western blot bands of IRF1, IRG1/CAD and β-ACTIN proteins are shown. (**D, E**) Metabolites were extracted from transfected cells after 4h of LPS stimulation and analysed by GC-MS. Itaconic acid levels in *Irf1* silenced cells were calculated as the percentage relative to the non-specific transfected cells in (**D**) control and (**E**) LPS activated cells. Error bars and statistical significance were calculated from 3 biological replicates (± SEM). *p < 0.05.

Silencing of *Irf1*, our first top scoring transcription factor, resulted in 55% and 45% decrease of the *Irf1* mRNA levels when compared to the non-targeting siRNA in unstimulated and LPS-treated cells, respectively (Fig 6A). Correspondingly, *Irg1* expression was significantly decreased by 66% and 26% in *Irf1* silenced cells as compared to non-targeting siRNA in unstimulated and LPS treated cells, respectively (Fig 6B). In parallel, proteins were extracted from the cells 4 hours after LPS stimulation. As expected, IRF1 and IRG1/CAD proteins were not detectable in control conditions, while they were detected in LPS-stimulated cells in both siRNA *Irf1* and non-targeting siRNA conditions. In accordance with gene expression results, IRF1 and IRG1/CAD expression levels both decreased in *Irf1* silenced cells as compared to non-targeting siRNA transfected cells following LPS stimulation (Fig 6C). As IRG1/CAD has been recently described to enzymatically convert cis-aconitic acid into itaconic acid [5], we next measured itaconic acid levels in *Irf1* silenced cells. Indeed, itaconic acid amounts in siRNA *Irf1* cells were decreased by 11% in unstimulated cells and by 20% in LPS-treated cells when compared to non-targeting siRNA treated cells (Fig 6D and 6E).

**Fig 7:**
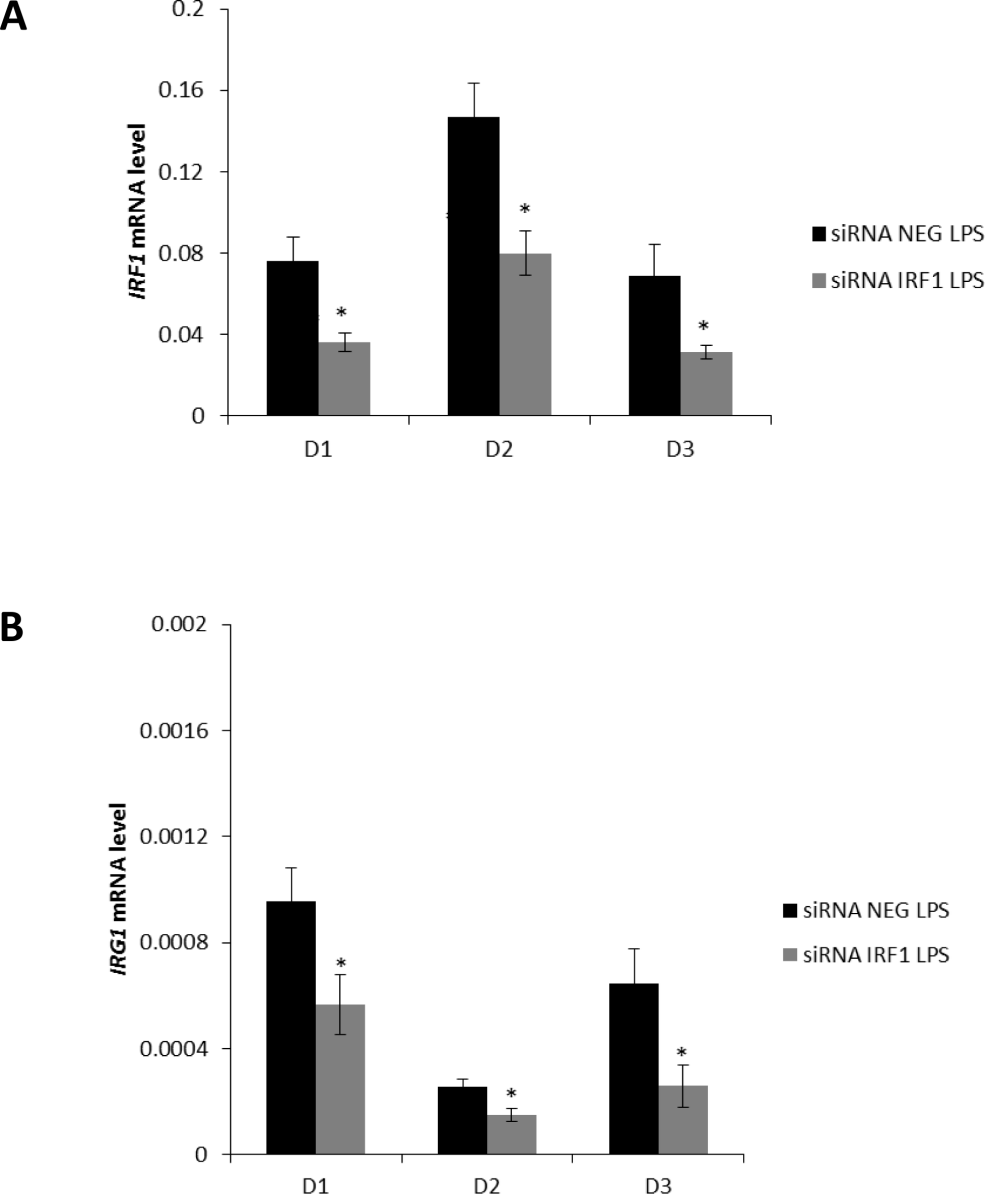
siRNA *IRF1* mediated gene silencing in human PBMCs-derived macrophages. PBMCs-derived macrophages were transfected with siRNA negative (siRNA NEG) or siRNA specific to *IRF1* (siRNA IRF1) 24 hours before treatment and RNA was extracted 6 hours after activation with LPS (10μg/ml) in independent donors (D1-D3). The bars show the mean of (**A**) *IRF1* and (**B**) *IRG1* mRNA levels of 3 technical replicates (± SEM) measured by real-time PCR normalised with L27 as the housekeeping gene. *p < 0.05.

Similarly to the mouse macrophages, human PBMCs-derived macrophages were transfected for 24 hours with siRNA specifically targeting *IRF1* or a non-targeting siRNA as control. Transfected macrophages were then stimulated with LPS and RNA was extracted after 6 hours. Differently from mouse macrophages, human primary macrophages do not have a detectable basal level of *IRG1* expression, thus we only analysed gene expression levels following LPS exposure. As a result of *IRF1* silencing, which held a decrease of its expression levels, as an average of four donors, by 54% (Fig 7A), *IRG1* expression levels were correspondingly reduced by approximately 50% under LPS conditions (Fig 7B) in siRNA *IRF1* transfected cells when compared to non-targeting siRNA treated cells. In order to gain additional support for IRF1 as a direct regulator of the *IRG1* locus, we overlaid the putative top scoring IRF1 binding motifs identified in our MATCH™ analysis (S2 Table) with open chromatin regions in the human *IRG1* locus in PBMCs and blood CD14+ monocytes using publicly available ENCODE data on UCSC Genome Browser (S8 Fig). The results show that, in the human monocyte lineage, IRF1 binding sites in the *IRG1* locus are associated with active chromatin marks, such as H3K4me1 and H3K27ac, which mark active/poised enhancers and which could represent putative IRF1 binding sites responsible for *IRG1* expression.

Taken together, these results show that IRF1 is a transcriptional regulator of *IRG1* in both mouse and human macrophages and that our combination of computational and experimental approaches represents an efficient method to identify transcriptional regulators of a given inducible gene.

## Discussion

We have recently revealed that *IRG1* codes for the enzyme IRG1/CAD which catalyses the decarboxylation of cis-aconitate, a TCA cycle intermediate, to itaconic acid [5]. This metabolite inhibits isocitrate lyase, the key enzyme of the glyoxylate shunt [35,36]. We demonstrated that silencing *Irg1* in murine macrophages decreases itaconic acid levels resulting in a defective antimicrobial activity towards different bacterial infections. Thus, we showed that IRG1/CAD, by the production of itaconic acid from a TCA cycle intermediate, plays an essential role in innate immune responses. Here, we additionally show that *IRG1* is not only expressed by human macrophages, but also by monocytes as well as by dendritic cells. Thus, due to the important role of IRG1/CAD at the interface between the innate immune system and the central carbon metabolism, we aimed to shed more light into its expression and transcriptional regulation under inflammatory conditions.

For this, we designed an integrative approach of computational and experimental methods to identify the transcriptional regulators of *IRG1* in mammalian macrophages, where the notion “co-expression implies co-regulation” is satisfied [37]. Transcription factors play a pivotal role in modulating gene expression and thus contribute to the overall regulation of biological processes. In order to identify the transcriptional regulators responsible for *IRG1* induction, we used a weight matrix-based tool for searching putative transcription factor binding sites in DNA sequences: MATCH^TM^ [25]. This tool uses the matrix library collected in TRANSFAC^®^, thus providing the possibility to search for a large variety of different transcription factor binding sites [25]. The main bottleneck in using these tools is the high amount of false positives generated due to the short and degenerate sequence motifs of transcription factors. A comprehensive method is hence needed to increase the specificity. With our algorithm, we eliminated non-cell type/condition specific transcription factors, thus rendering the biological validation appropriate. Though our method could potentially identify the transcriptional regulators of any given gene, it presents some limitations. For instance, to contextualize our networks, we exclusively considered DEGs from microarrays data, although some transcription factors are phosphorylated in order to be activated and shuttled into the nucleus to subsequently act as transcription factors. Thus, in follow-up studies it would be valuable to add an additional layer of information on top of the transcriptomic data, such as phosphoproteomics data. This could allow contextualizing the generated networks not only taking into account gene expression data, but also the phosphorylation data of various regulating proteins.

From our contextualized murine and human networks, we identified potential transcriptional regulators of *IRG1* in macrophages. Of interest, our mouse and human analysis provide insights for the identification of species-specific regulators for *IRG1* induction under LPS activation. Although the human data were obtained from primary cells, while the mouse analysis was conducted using the macrophage cell line RAW264.7, it is tempting to speculate that the transcriptional machinery inducing *IRG1* expression, with the exception of IRF1, is mostly species-specific, as highlighted by the different transcriptional regulators identified in the two species (Table 1). Experimentally, we confirmed that CEBPB in mouse and IRF1 in both species represent positive targets which modulate *IRG1* expression. We used CEBPB as a proof of concept for our method, since we predicted it in our ranking and it has been previously shown to be a transcriptional regulator of *irg1* in zebrafish (*Danio rerio*). CEBPB, as a primary response gene, is known as a transcriptional regulator of the acute phase response [38]. In our experimental conditions in murine macrophages, *Cebpb* is highly up-regulated under LPS stimulation and, when silenced, *Irg1* expression levels are decreased. These results are in agreement with those obtained in zebrafish, thus highlighting CEBPB as a transcriptional regulator of *Irg1* in both the species.

IRF1 is referred to as a positive transcription factor as it induces several genes, which are important in regulating several immunological and physiological functions in mammalian cells. *Irf1* is also transcriptionally activated by several pro-inflammatory cytokines and pathogens [39,40]. IRF1 selectively regulates specific sets of genes depending on the cell type and the appropriate response needed to counter the external stimuli, thus having its function driven by cell-type specific factors. IRF1 mainly activates interferons (IFNs) and IFN inducible genes. Some of the targets of IRF1 which are known to play a role in host defence are IFN (α, β), inducible nitric oxide synthase (iNOS), interleukin 12 (IL12), cyclooxygenase 2 (COX2), IL15, caspase 1–7 and lysyl oxidase [17,41]. Among these, *iNOS* is one of the most studied downstream targets of IRF1. The enzyme iNOS catalyses the production of nitric oxide (NO) which is essential for the antimicrobial and anti-tumorigenic properties of macrophages. NO is a biologically active intermediate produced by the cells using L-arginine, an urea cycle intermediate, as the substrate [17,42]. Increased NO production correlates with resistance towards invading pathogens [43]. Several studies showed that the silencing of *Irf1* diminishes the expression of *iNOS* and the production of NO, thereby attenuating the antibacterial and antiviral activities and thus worsening the severity of the disease [43–45]. *iNOS* is induced upon stimulation by IL1, IL12, TNF, IFNγ and LPS via IRF1 [46]. Nevertheless, IFNγ and LPS are the major and necessary stimulants to induce *iNOS* expression and NO production in murine macrophages [47]. Its transcriptional induction following LPS stimulation occurs after the synthesis of IFNs and the JAK/STAT signalling pathway [48]. *iNOS* promoter requires the dimeric binding of IRF1 for the full cytokine activation. Hence, other transcription factors, like NFκB, that are induced by other cytokines, may cooperate with IRF1 to induce the full transcriptional activity of *iNOS* [49]. IRF1 protein is highly unstable with a half-life of 30 minutes [50] and LPS-induced NO production can be abrogated by inhibiting the translation of *Irf1* by 10-hydroxy-*trans*-2-decenoic acid, a medium chain fatty acid [51]. IRF1 is serine-phosphorylated by protein kinase A (PKA) and protein kinase C (PKC). Mutation of these tyrosine residues inhibit the induction of *Irf1* expression [52,53]. It was reported earlier that LPS induction of *Irg1* in macrophages is mediated via the PKC pathway [3]. Thus the PKC pathway may be responsible for *Irg1* induction through IRF1.

*Irg1* was shown to be co-regulated and co-expressed with *iNOS* when murine cells were either infected with *Mycobacterium* [13] or stimulated with LPS and was also reported as a family member of IFN inducible genes [14,54]. Of interest, Mycobacteria infection studies in IRF1 knock-out mice revealed that IRF1 is required for the mycobacteria induced granuloma necrosis. Gene expression analysis of infected mice resulted in the grouping of differentially expressed genes into different clusters based on the dependence of gene expression on IFNγ or IRF1. *Irg1* was classified to the cluster of genes whose expression is mostly dependent on *Irf1*, but less on IFNγ [55]. In line with the results obtained with Mycobacteria infections, our results show that *IRF1* expression is highly up-regulated under LPS exposure and that *IRF1* silencing induces a significant decrease of *IRG1* expression levels in both human and murine macrophages. Upon TLR activation by external stimuli, such as LPS, IRF1 was shown to interact with MYD88 adaptor molecule to translocate into the nucleus and induce the expression of several genes to mediate immune responses [56]. The analysis of transcription start site (TSS) distributions of immune related genes by Liang *et al*. classified *Irf1* as a gene dependent on MYD88 pathway along with *Irg1* which was stated as an interferon stimulated gene (ISG) element [57]. However, *Irg1* was previously shown to be expressed independently from MYD88 or TRIF adaptor molecules [14,15], thus demonstrating that additional pathways regulating *Irg1* expression independent from IRF1 and MYD88 could exist.

It is already known that *IRF1* has a significant role in antibacterial and antiviral responses, induction of apoptosis and tumorigenesis, with several of these processes known to be mediated by iNOS and NO [58]. Thus, from our results, it is tempting to conclude that IRF1 regulates these processes also through the induction of *Irg1* and itaconic acid production. To this end, *iNOS* and *IRG1* can be considered as the gene twins, regulated by IRF1, which contribute to the host protection towards pathogen invasion through the production of effector molecules, such as NO and itaconic acid, respectively. Of interest, in contrast to mouse, *iNOS* expression and NO production in human cellular systems have been an argument of discussion by cellular biologists and immunologists since a long time [59]. Even though there were reports showing low levels of *iNOS* and NO in activated human cells [60–62], it was argued that these results neither show the functional pathway of NO production, as through L-arginine in mouse, nor describe the precise experimental details, such as cell types and culture conditions [63–65]. In accordance with this argument, accordingly to our microarrays data, we did not detect *iNOS* expression in LPS-stimulated human macrophages, while its expression was highly up-regulated in mouse cells. However, taking the side of the discussion that human macrophages do not express iNOS, IRF1 plays a crucial role in mediating antimicrobial responses through *IRG1* gene regulation in human immune cells.

Finally, in a recent study *Irf1* and *Irg1* expression levels were correlated when neurons were infected with several viruses such as Saint Louis encephalitis virus (SLEV) and a coronavirus (mouse hepatitis virus, MHV). *Irg1* had higher basal and also IFN-β–induced expression levels in granule cell neurons when compared to cortical neurons. Viral replication was increased in granule cell neurons when transduced with lentiviruses expressing shRNA targeting *Irg1* [66], thus postulating the role of *Irg1* in inhibiting viral replication in neurons. *Irf1* induction by interferons is already known as an antiviral response against certain viruses [45]. Even though the role of *Irg1* in antiviral responses is not yet reported, future studies could reveal that IRF1 mediates antiviral actions through the induction of *Irg1*.

## Conclusion

Here we provide evidence for a method integrating computational and experimental approaches as a tool to successfully identify transcriptional regulators of a given inducible gene, thereby stepping towards the understanding of its gene regulatory network.

Improved knowledge about *IRG1* transcriptional regulation provides a better understanding of the molecular mechanisms that are involved in specific inflammatory and infectious diseases. To this end, we identified IRF1 as a transcriptional regulator of *IRG1* expression in both human and mouse macrophages under inflammatory conditions. Understanding the mechanisms of *IRG1* induction during immune responses could lead to novel therapeutic approaches, which aim to modulate the intrinsic host response.

## Supporting Information

S1 Fig Time course of *IRG1* expression in human immune cells under LPS activation. RNA was extracted from (**A**) PBMCs-derived monocytes, (**B**) PBMCs-derived macrophages at 6, 9, 12, 15, 18, 21 and 24 hours after treatment with LPS (10μg/ml) in independent donors (D5-D8). Time points 12h and 21h were not recorded in D7 and D8 donors. The bars show the mean of 3 technical replicates (± SEM) of *IRG1* mRNA levels measured by real-time PCR normalised with L27 as the housekeeping gene. ***p < 0.001, **p < 0.01, *p < 0.05.
(TIF)

S2 Fig Short time course of *IRG1* expression in human and mouse macrophages under LPS activation. (**A**) RNA was extracted from PBMCs-derived macrophages at 20, 40, 60, 80, 100, 120 minutes as well as at 3, 4 and 5 hours after treatment with LPS (10μg/ml). The bars show the mean of 3 technical replicates (± SEM) of *IRG1* mRNA levels measured by real-time PCR normalised with L27 as the housekeeping gene. ***p < 0.001, *p < 0.05. **B**) RNA was extracted from RAW264.7 macrophages at 20, 40, 60, 80, 100, 120 minutes after treatment with LPS (10ng/ml). The bars show the mean of 3 biological replicates (± SEM) of *Irg1* mRNA levels measured by real-time PCR normalised with L27 as the housekeeping gene. ***p < 0.001, **p < 0.01, *p < 0.05.
(TIF)

S3 Fig Heatmap and Gene Ontology (GO) analysis (Biological processes) of mouse macrophages under LPS stimulation. (**A**) Heatmap showing the top 100 differentially expressed genes (log2 FC≥1 and p-value <0.01) between control and 6 hours LPS-activated (10ng/ml) mouse RAW264.7 macrophages. Individual biological replicates are shown as individual columns for control (n = 3) and LPS (n = 3). Relative expression levels are shown from low (green) to high (red). (**B, C**) GO biological processes that are significantly represented by (**B**) up-regulated genes and (**C**) down-regulated genes.
(TIF)

S4 Fig Heatmap and Gene Ontology (GO) analysis (Biological processes) of human macrophages under LPS stimulation. (**A**) Heatmap showing the top 100 differentially expressed genes (log2 FC≥1 and p-value <0.01) between control and 6 hours LPS-activated (10μg/ml) human PBMCs-derived macrophages. Individual biological replicates are shown as individual columns for control (n = 3) and LPS (n = 3). Relative expression levels are shown from low (green) to high (red). (**B, C**) GO biological processes that are significantly represented by (**B**) up-regulated genes and (**C**) down-regulated genes.
(TIF)

S5 Fig Contextualised gene regulatory network of mouse RAW264.7 macrophages. Gene regulatory network (GRN) obtained after the contextualisation of the merged GRN (LPS downstream and *Irg1* upstream) with the booleanised gene expression data of mouse RAW264.7 macrophages (LPS vs Control). The hierarchical layout of the network was created using CytoScape. The genes (nodes) in red are upregulated and in green are downregulated in LPS stimulated RAW264.7 macrophages. The interactions (edges) with solid lines are activations and the dashed line edges are inhibitions. *Irg1* and LPS (blue coloured) are highlighted with a dark circle. The following are the nodes from the mouse network: 1-NOD2, 2-RIPK2, 3-SOCS3, 4-CXCR4, 5-CASP1, 6-IL1A, 7-IL1B, 8-IL18, 9-CCL2, 10-CCRL2, 11-TNF, 12-FAS, 13-LPS, 14-CD80, 15-IRAK3, 16-NOS2, 17-IL12B, 18-CASP4, 19-PSMB9, 20-PTGS2, 21-IRF7, 22-IL6, 23-CD40, 24-NFKBIZ, 25-IRF1, 26-TAP1, 27-IRF9, 28-STAT1, 29-SOCS1, 30-PRDM1, 31-JUNB, 32-CEBPD, 33-CEBPB, 34-CSF3, 35-IRG1, 36-ZFP36, 37-PTGES, 38-PTGS1, 39-EMR1, 40-LC40A.
(TIF)

S6 Fig Contextualised gene regulatory network of human PBMCs-derived macrophages. Gene regulatory network (GRN) obtained after the contextualisation of the merged GRN (LPS downstream and *IRG1* upstream) with the booleanised gene expression data of human monocyte-derived macrophages (LPS vs Control). The hierarchical layout of the network was created using CytoScape. The genes (nodes) in red are upregulated and in green are downregulated in LPS stimulated PBMCs-derived macrophages. The interactions (edges) with solid lines are activations and the dashed line edges are inhibitions. *IRG1* and LPS (blue coloured) are highlighted with a dark circle. The following are the nodes from the human network: 1-CD226, 2-CSF1R, 3-CXCL2, 4-CXCR4, 5-SOCS3, 6-STAT5A, 7-TNFRSF21, 8-AHR, 9-CTSD, 10-VDR, 11-NFKB1, 12-IRF1, 13-STAT4, 14-RARA, 15-ETS2, 16-RUNX1, 17-IRG1, 18-NFKBIZ, 19-LPS, 20-FOS, 21-IL8, 22-CCL2, 23-CCR2, 24-SOAT1, 25-KLF4, 26-TCF7L2, 27-KLF8, 28-CCND1, 29-MYC, 30-ECM1, 31-PLSCR1, 32-NR1H3, 33-APOE.
(TIF)

S7 Fig siRNA *Cebpb* mediated gene silencing in mouse RAW264.7 macrophages. RAW264.7 cells were transfected with siRNA negative (siRNA NEG) or siRNA specific to *Cebpb* (siRNA Cebpb) 24 hours before treatment and RNA was extracted 2 hours after activation with LPS (10ng/ml). The bars show the mean of 3 biological replicates (± SEM) of (**A**) *Cebpb* and (**B**) *Irg1* mRNA levels measured by real-time PCR normalised with L27 as the housekeeping gene. *p < 0.05.
(TIF)

S8 Fig Visualization of putative IRF1 binding sites at the human *IRG1* locus. Top scoring putative IRF1 binding motifs identified in our MATCH™ analysis overlaid with chromatin regions in the human *IRG1* locus in PBMCs and blood CD14+ monocytes using publicly available ENCODE data on UCSC Genome Browser.
(TIF)

S1 Table List of primers used in RT-PCR analysis. (XLSX)

S2 Table Predicted binding sites on human and mouse *IRG1* promoter which have both the Core and Matrix similarity Scores ≥ 0.90 as given by MATCH™ algorithm. (XLSX)

S3 Table Differentially expressed genes (DEGs) with their respective log fold changes and p-values in mouse RAW264.7 macrophages activated with LPS (logFC ≥ |1|, p<0.01). (XLSX)

S4 Table Differentially expressed genes (DEGs) with their respective log fold changes and p-values in PBMCs-derived macrophages activated with LPS (logFC ≥ |1|, p<0.01). (XLSX)
