# Supplementary figures and images for: Gene Regulatory Network Inference of Immunoresponsive Gene 1 (IRG1) Identifies Interferon Regulatory Factor 1 (IRF1) as Its Transcriptional Regulator in Mammalian Macrophages
Source: PLoS One. 2016 Feb 12;11(2):e0149050. doi: 10.1371/journal.pone.0149050 (PMC4752512; doi:10.1371/journal.pone.0149050)

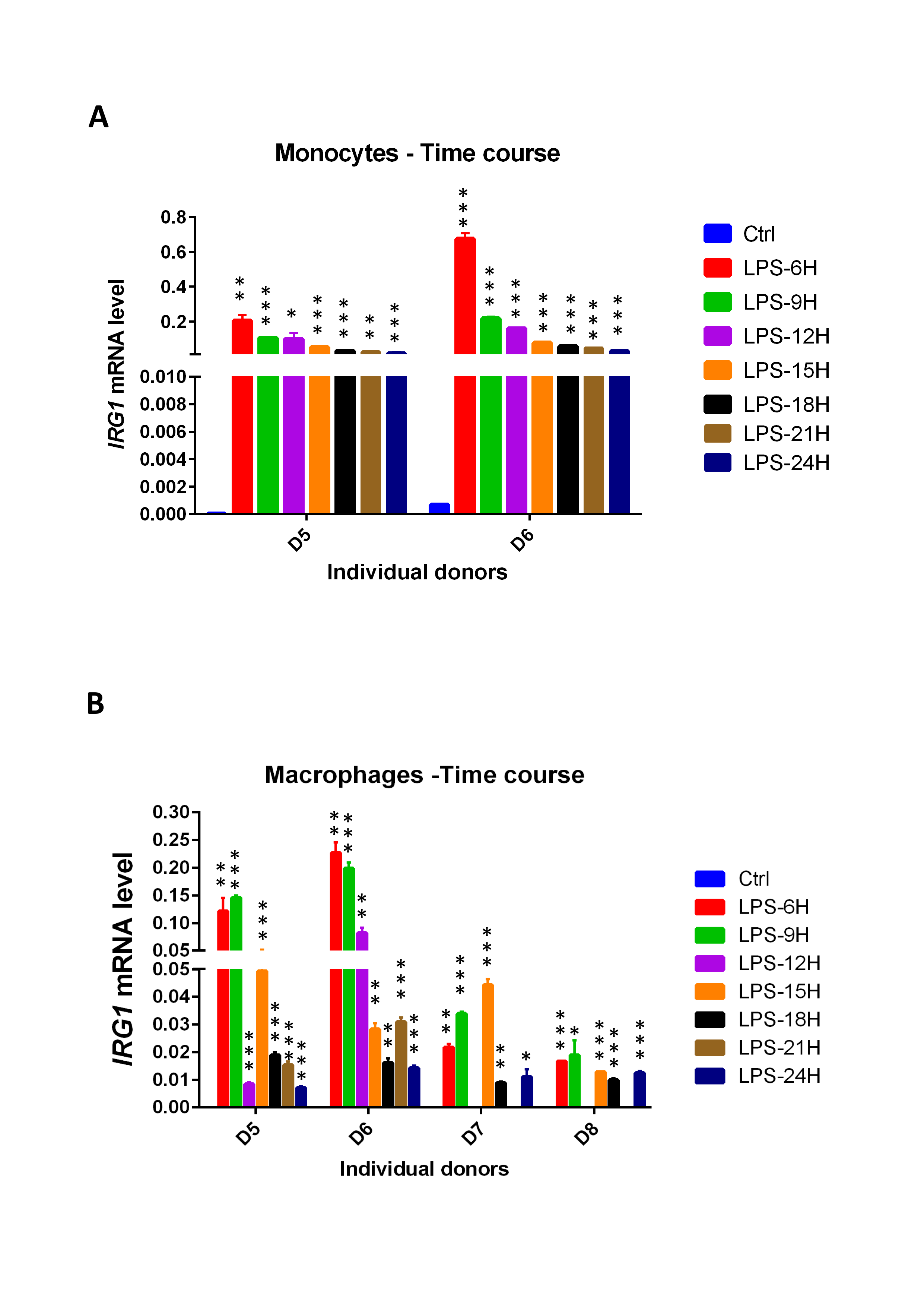

Supplement: S1 Fig — RNA was extracted from (A) PBMCs-derived monocytes, (B) PBMCs-derived macrophages at 6, 9, 12, 15, 18, 21 and 24 hours after treatment with LPS (10μg/ml) in independent donors (D5-D8). Time points 12h and 21h were not recorded in D7 and D8 donors. The bars show the mean of 3 technical replicates (± SEM) of IRG1 mRNA levels measured by real-time PCR normalised with L27 as the housekeeping gene. ***p < 0.001, **p < 0.01, *p < 0.05. (TIF) [file pone.0149050.s001.tif]

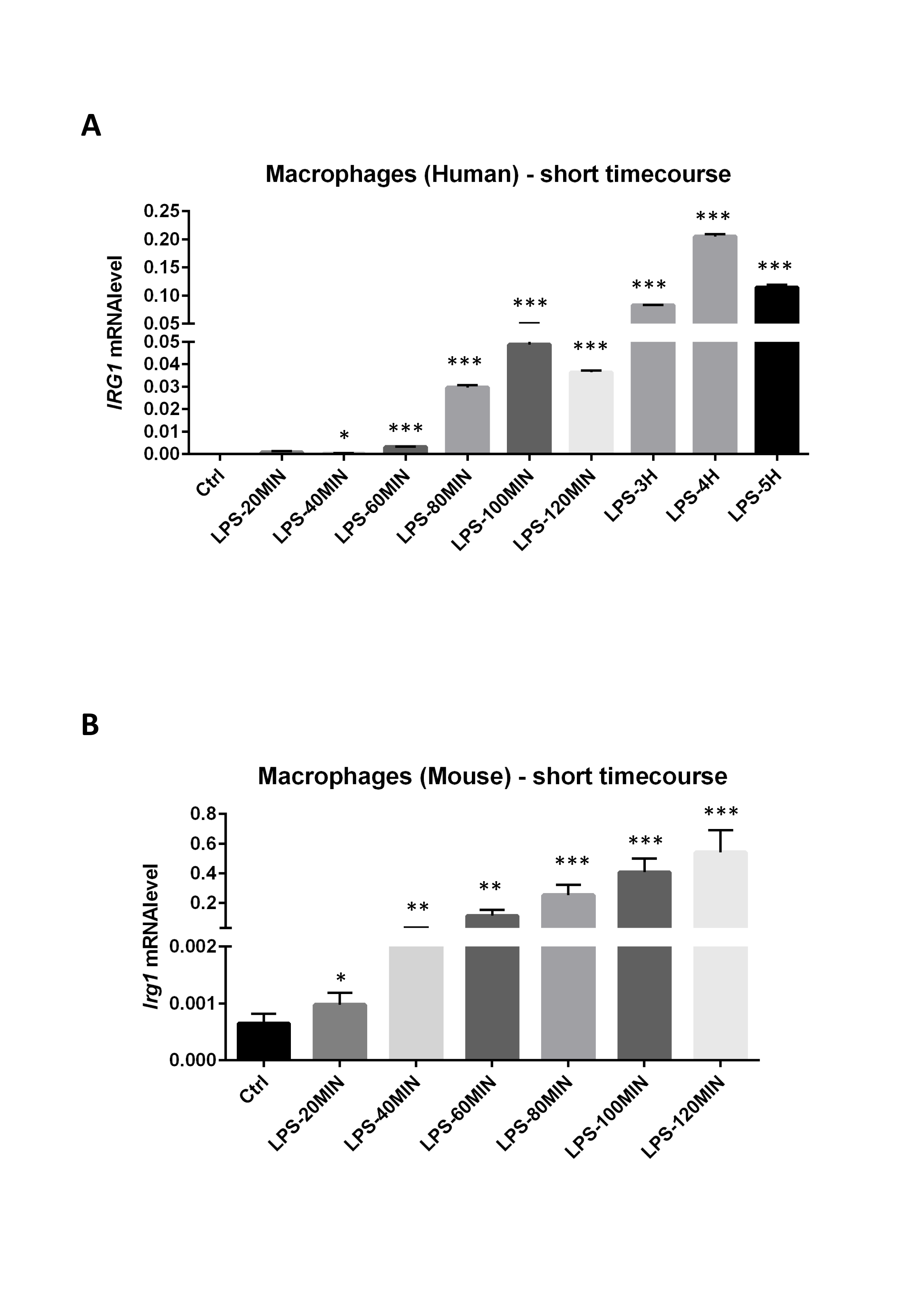

Supplement: S2 Fig — (A) RNA was extracted from PBMCs-derived macrophages at 20, 40, 60, 80, 100, 120 minutes as well as at 3, 4 and 5 hours after treatment with LPS (10μg/ml). The bars show the mean of 3 technical replicates (± SEM) of IRG1 mRNA levels measured by real-time PCR normalised with L27 as the housekeeping gene. ***p < 0.001, *p < 0.05. B) RNA was extracted from RAW264.7 macrophages at 20, 40, 60, 80, 100, 120 minutes after treatment with LPS (10ng/ml). The bars show the mean of 3 biological replicates (± SEM) of Irg1 mRNA levels measured by real-time PCR normalised with L27 as the housekeeping gene. ***p < 0.001, **p < 0.01, *p < 0.05. (TIF) [file pone.0149050.s002.tif]

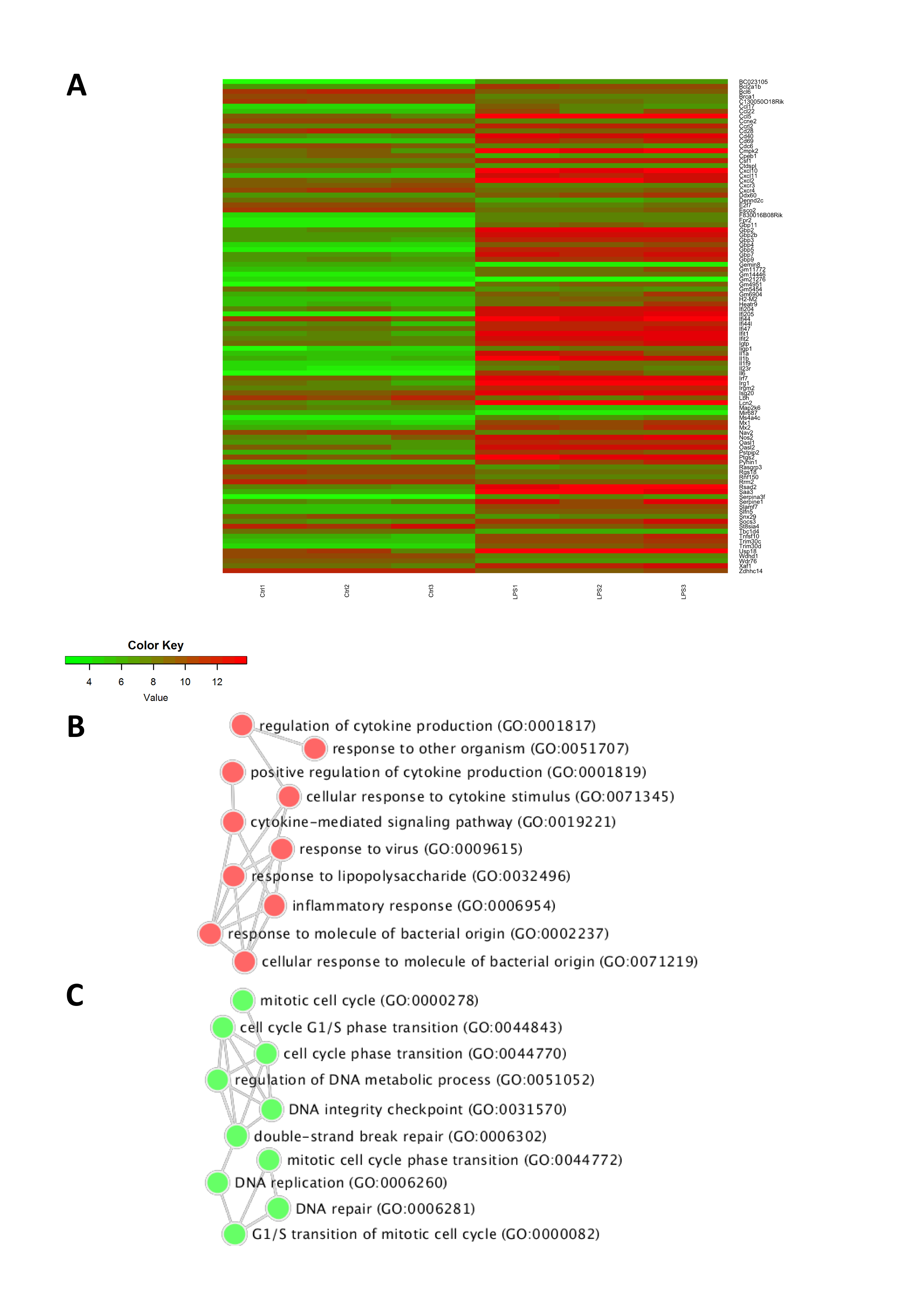

Supplement: S3 Fig — (A) Heatmap showing the top 100 differentially expressed genes (log2 FC≥1 and p-value <0.01) between control and 6 hours LPS-activated (10ng/ml) mouse RAW264.7 macrophages. Individual biological replicates are shown as individual columns for control (n = 3) and LPS (n = 3). Relative expression levels are shown from low (green) to high (red). (B, C) GO biological processes that are significantly represented by (B) up-regulated genes and (C) down-regulated genes. (TIF) [file pone.0149050.s003.tif]

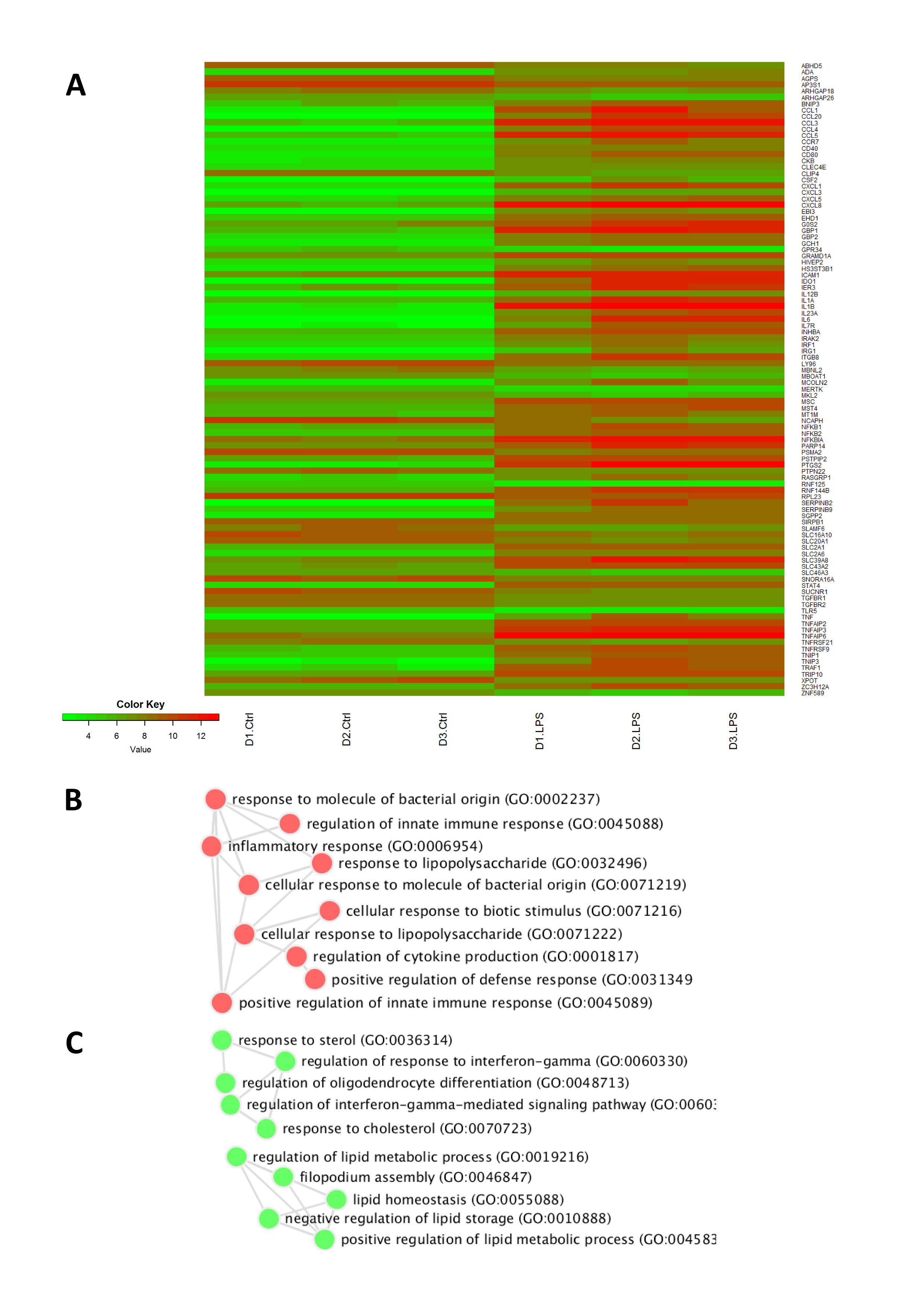

Supplement: S4 Fig — (A) Heatmap showing the top 100 differentially expressed genes (log2 FC≥1 and p-value <0.01) between control and 6 hours LPS-activated (10μg/ml) human PBMCs-derived macrophages. Individual biological replicates are shown as individual columns for control (n = 3) and LPS (n = 3). Relative expression levels are shown from low (green) to high (red). (B, C) GO biological processes that are significantly represented by (B) up-regulated genes and (C) down-regulated genes. (TIF) [file pone.0149050.s004.tif]

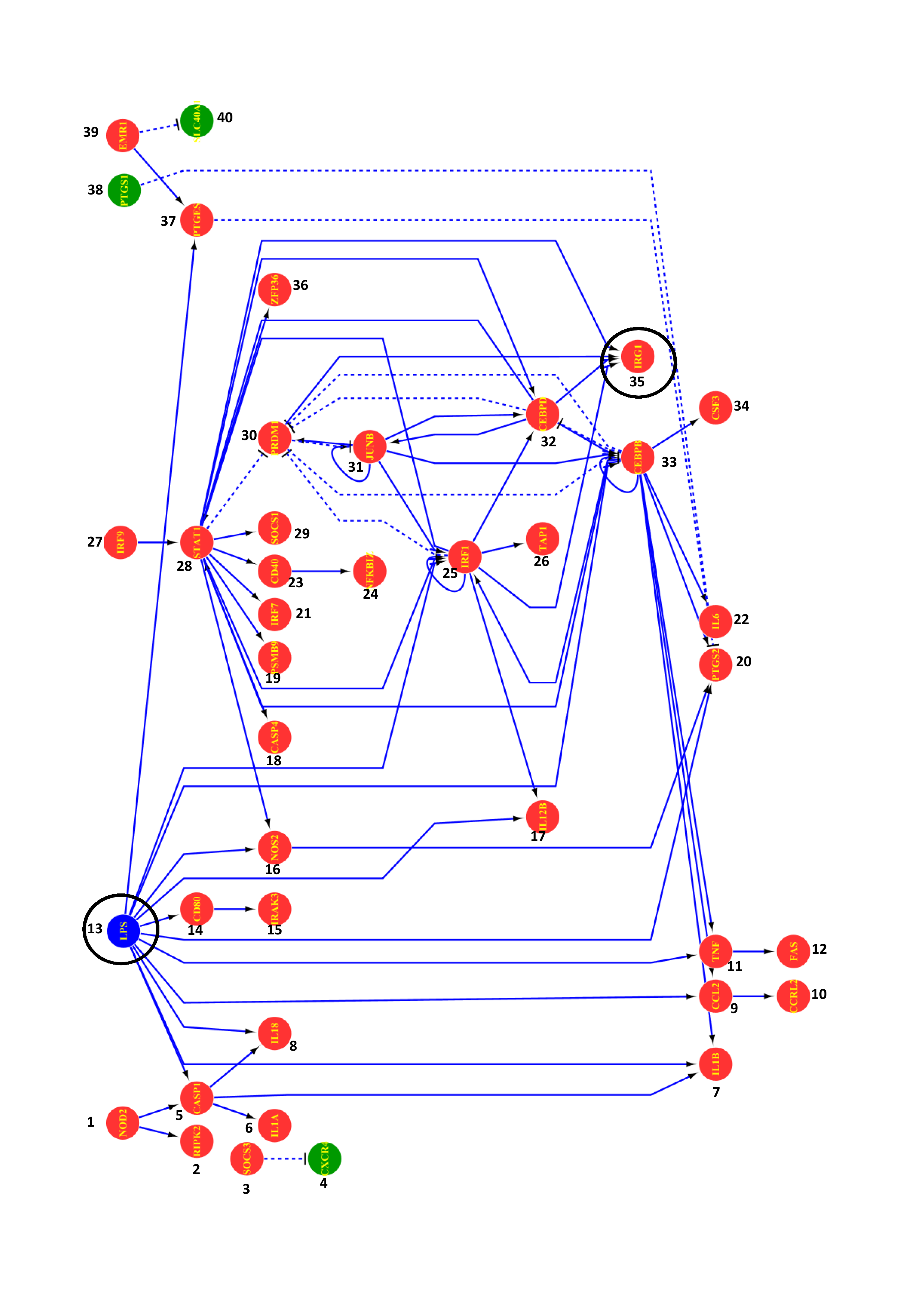

Supplement: S5 Fig — Gene regulatory network (GRN) obtained after the contextualisation of the merged GRN (LPS downstream and Irg1 upstream) with the booleanised gene expression data of mouse RAW264.7 macrophages (LPS vs Control). The hierarchical layout of the network was created using CytoScape. The genes (nodes) in red are upregulated and in green are downregulated in LPS stimulated RAW264.7 macrophages. The interactions (edges) with solid lines are activations and the dashed line edges are inhibitions. Irg1 and LPS (blue coloured) are highlighted with a dark circle. The following are the nodes from the mouse network: 1-NOD2, 2-RIPK2, 3-SOCS3, 4-CXCR4, 5-CASP1, 6-IL1A, 7-IL1B, 8-IL18, 9-CCL2, 10-CCRL2, 11-TNF, 12-FAS, 13-LPS, 14-CD80, 15-IRAK3, 16-NOS2, 17-IL12B, 18-CASP4, 19-PSMB9, 20-PTGS2, 21-IRF7, 22-IL6, 23-CD40, 24-NFKBIZ, 25-IRF1, 26-TAP1, 27-IRF9, 28-STAT1, 29-SOCS1, 30-PRDM1, 31-JUNB, 32-CEBPD, 33-CEBPB, 34-CSF3, 35-IRG1, 36-ZFP36, 37-PTGES, 38-PTGS1, 39-EMR1, 40-LC40A. (TIF) [file pone.0149050.s005.tif]

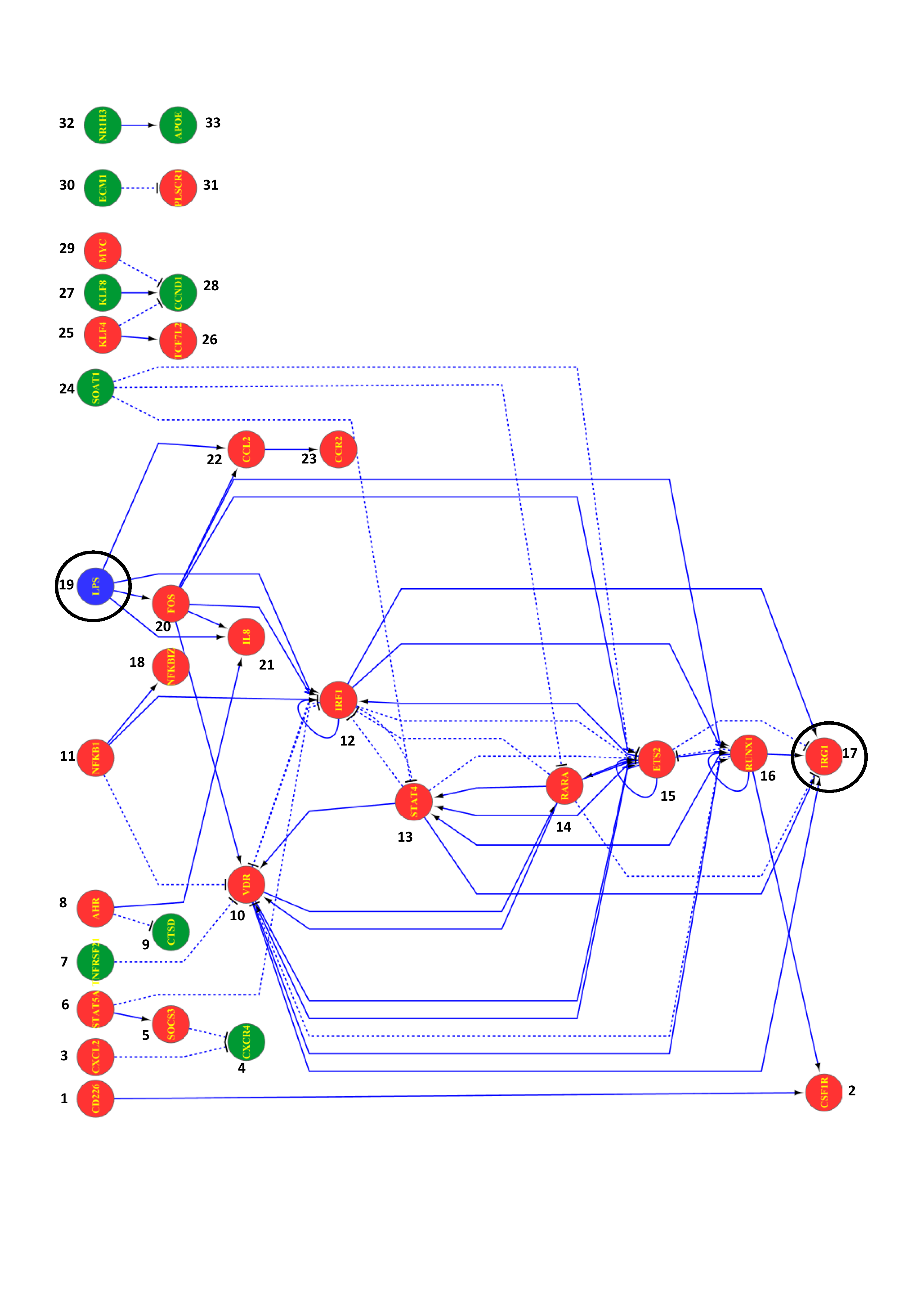

Supplement: S6 Fig — Gene regulatory network (GRN) obtained after the contextualisation of the merged GRN (LPS downstream and IRG1 upstream) with the booleanised gene expression data of human monocyte-derived macrophages (LPS vs Control). The hierarchical layout of the network was created using CytoScape. The genes (nodes) in red are upregulated and in green are downregulated in LPS stimulated PBMCs-derived macrophages. The interactions (edges) with solid lines are activations and the dashed line edges are inhibitions. IRG1 and LPS (blue coloured) are highlighted with a dark circle. The following are the nodes from the human network: 1-CD226, 2-CSF1R, 3-CXCL2, 4-CXCR4, 5-SOCS3, 6-STAT5A, 7-TNFRSF21, 8-AHR, 9-CTSD, 10-VDR, 11-NFKB1, 12-IRF1, 13-STAT4, 14-RARA, 15-ETS2, 16-RUNX1, 17-IRG1, 18-NFKBIZ, 19-LPS, 20-FOS, 21-IL8, 22-CCL2, 23-CCR2, 24-SOAT1, 25-KLF4, 26-TCF7L2, 27-KLF8, 28-CCND1, 29-MYC, 30-ECM1, 31-PLSCR1, 32-NR1H3, 33-APOE. (TIF) [file pone.0149050.s006.tif]

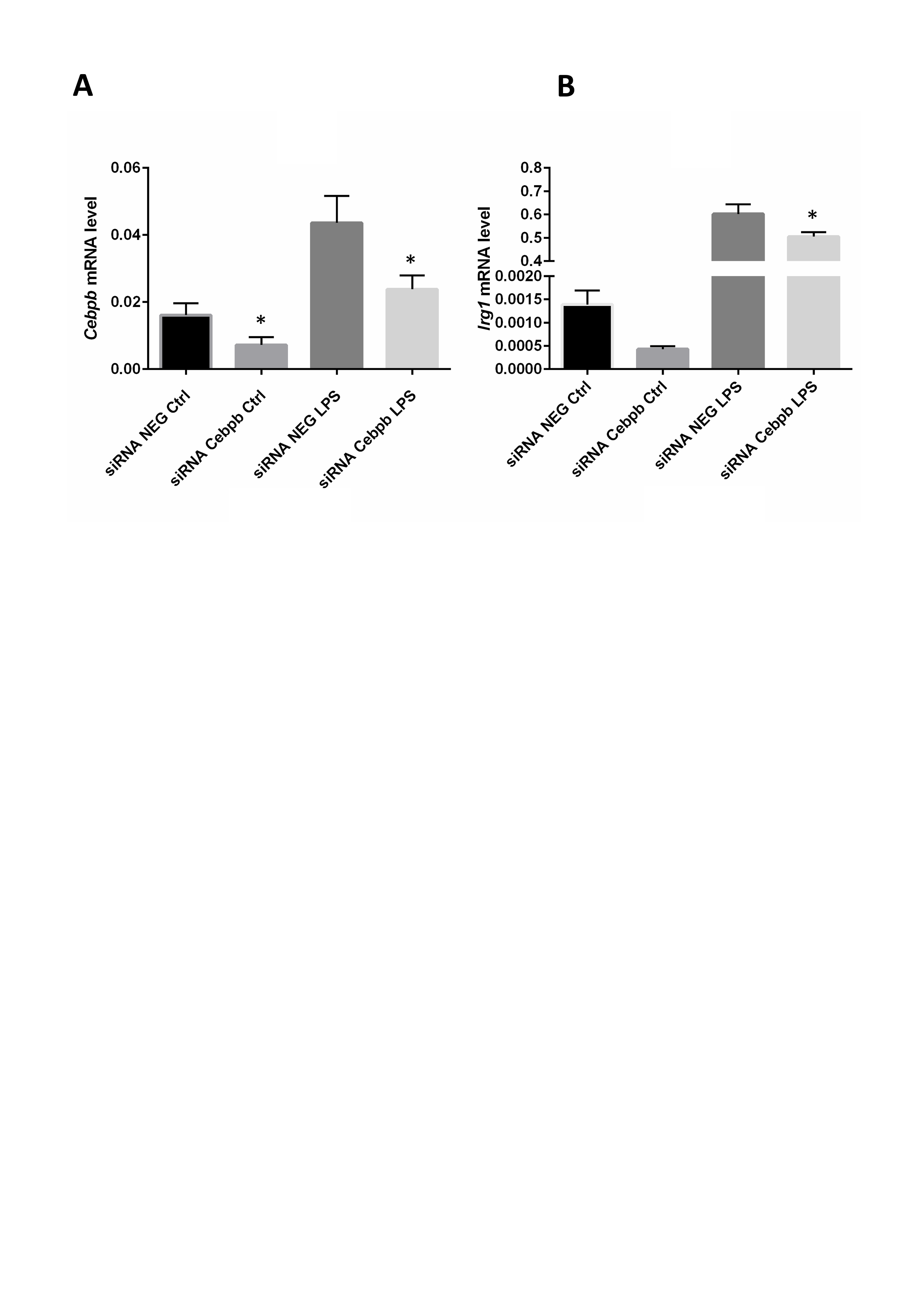

Supplement: S7 Fig — RAW264.7 cells were transfected with siRNA negative (siRNA NEG) or siRNA specific to Cebpb (siRNA Cebpb) 24 hours before treatment and RNA was extracted 2 hours after activation with LPS (10ng/ml). The bars show the mean of 3 biological replicates (± SEM) of (A) Cebpb and (B) Irg1 mRNA levels measured by real-time PCR normalised with L27 as the housekeeping gene. *p < 0.05. (TIF) [file pone.0149050.s007.tif]

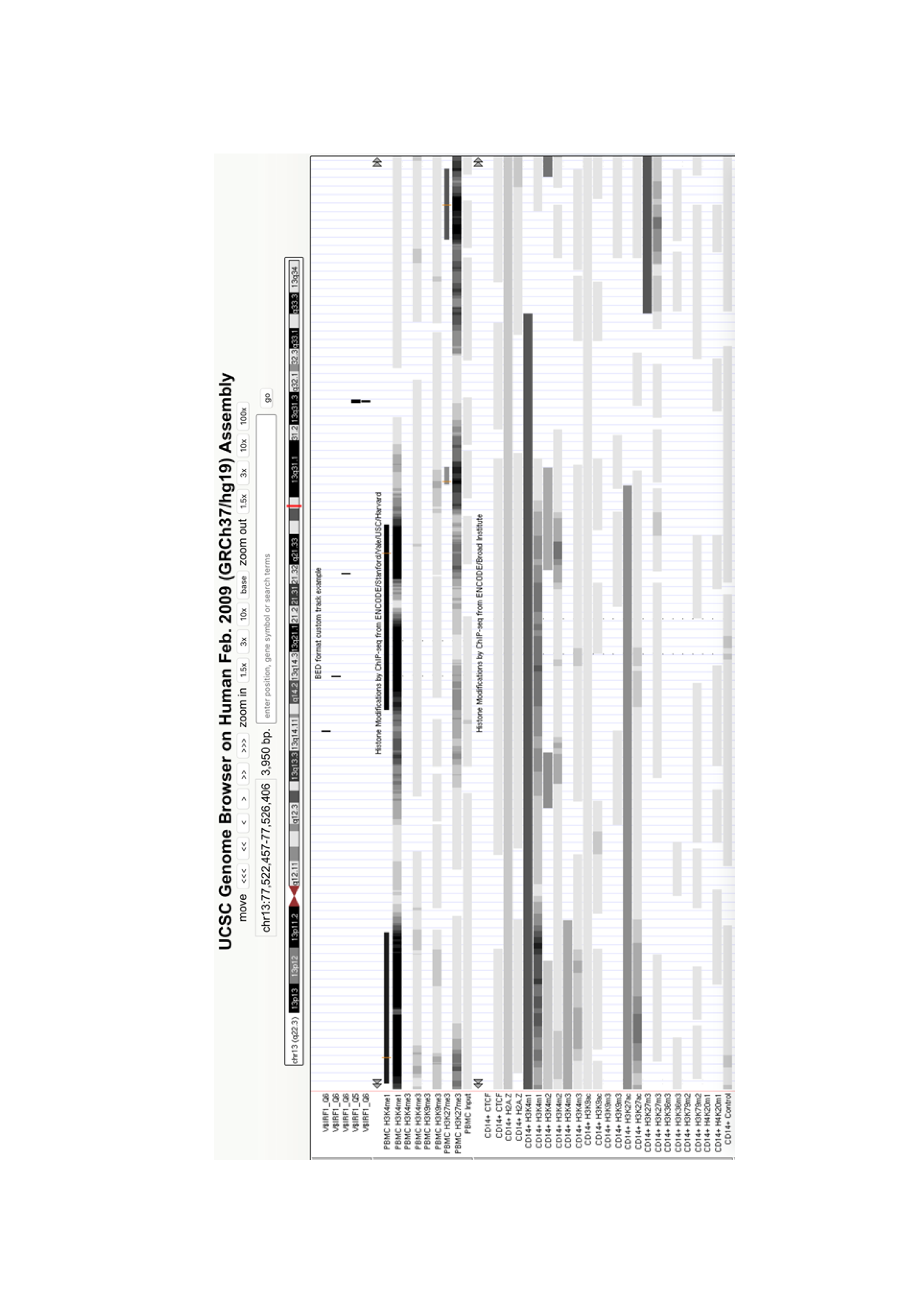

Supplement: S8 Fig — Top scoring putative IRF1 binding motifs identified in our MATCH™ analysis overlaid with chromatin regions in the human IRG1 locus in PBMCs and blood CD14+ monocytes using publicly available ENCODE data on UCSC Genome Browser. (TIF) [file pone.0149050.s008.tif]
